# HDAC inhibitors suppress c-Jun/Fra-1-mediated proliferation through transcriptionally downregulating MKK7 and Raf1 in neuroblastoma cells

**DOI:** 10.18632/oncotarget.6797

**Published:** 2015-12-30

**Authors:** Weiwen He, Yanna Wu, Xiaomei Tang, Yong Xia, Guozhen He, Zhiqun Min, Chun Li, Shiqiu Xiong, Zhi Shi, Yongjian Lu, Zhongmin Yuan

**Affiliations:** ^1^ Department of Neurosurgery, The Second Affiliated Hospital of Guangzhou Medical University, Guangzhou, China; ^2^ Institute of Neuroscience, Key Laboratory of Neurogenetics and Channelopathies of Guangdong Province and Ministry of Education of China, Guangzhou Medical University, Guangzhou, China; ^3^ Clinical Laboratory Center of Molecular Medicine, The Second Affiliated Hospital of Guangzhou Medical University, Guangzhou, China; ^4^ Department of Biochemistry, University of Leicester, Leicester, UK; ^5^ Department of Cell Biology and Institute of Biomedicine, College of Life Science and Technology, Jinan University, Guangzhou, China

**Keywords:** neuroblastoma, Fra-1/c-Jun dimer, MKK7, HDAC inhibitor, proliferation

## Abstract

Activator protein 1 (AP-1) is a transcriptional factor composed of the dimeric members of bZIP proteins, which are frequently deregulated in human cancer cells. In this study, we aimed to identify an oncogenic AP-1 dimer critical for the proliferation of neuroblastoma cells and to investigate whether histone deacetylase inhibitors (HDACIs), a new generation of anticancer agents, could target the AP-1 dimer. We report here that HDACIs including trichostatin A, suberoylanilidehydroxamic acid, valproic acid and M344 can transcriptionally suppress both c-Jun and Fra-1, preceding their inhibition of cell growth. c-Jun preferentially interacting with Fra-1 as a heterodimer is responsible for AP-1 activity and critical for cell growth. Mechanistically, HDACIs suppress Fra-1 expression through transcriptionally downregulating Raf1 and subsequently decreasing MEK1/2-ERK1/2 activity. Unexpectedly, HDACI treatment caused MKK7 downregulation at both the protein and mRNA levels. Deletion analysis of the 5′-flanking sequence of the MKK7 gene revealed that a major element responsible for the downregulation by HDACI is located at −149 to −3 relative to the transcriptional start site. Knockdown of MKK7 but not MKK4 remarkably decreased JNK/c-Jun activity and proliferation, whereas ectopic MKK7-JNK1 reversed HDACI-induced c-Jun suppression. Furthermore, suppression of both MKK-7/c-Jun and Raf-1/Fra-1 activities was involved in the tumor growth inhibitory effects induced by SAHA in SH-SY5Y xenograft mice. Collectively, these findings demonstrated that c-Jun/Fra-1 dimer is critical for neuroblastoma cell growth and that HDACIs act as effective suppressors of the two oncogenes through transcriptionally downregulating MKK7 and Raf1.

## INTRODUCTION

Neuroblastoma (NB), which is typically derived from neural crest tissues of the sympathetic nervous system, accounts for approximately 10% of all childhood cancers and 50% of childhood cancer deaths [1]. Amplification of the MYCN is the predominant marker for aggressive NB and correlates with poor prognosis, found in 20% cases of NBs [2]. Approximately half of all cases are currently classified as highrisk for disease relapse, with overall survival rates of less than 40%, despite intensive multimodal therapy. As NBs that are not curable with current therapeutic modalities commonly exhibit defects in growth arrest and apoptosis [3], identifying the factors that play critical roles in driving NB malignant transformation or progression, as well as promoting survival and proliferation is thus particularly attractive for developing therapeutic targets.

AP-1 proteins are composed of members with a basic leucine zipper (bZIP) domain, including the Jun (c-Jun, JunB and JunD) and Fos (c-Fos, FosB, Fra-1 and Fra-2) protein families, Jun dimerization partners (JDP1 and JDP2), and the activation transcription factor (ATF; ATF2, LRF1/ATF3 and B-ATF) and MAF subfamilies. Forming stable homodimers or heterodimers among AP-1 proteins is essential for their functions in transcription and in binding to conserved cis-elements, e.g., the 12-O-tetradecanoylphorbol-13-acetate (TPA) responsive element (TRE, 5′-TGAG/CTCA-3′) and cAMP responsive element (CRE, 5′-TGACGTCA-3′), through which a variety of genes involved in cellular proliferation and apoptosis are regulated [4]. Moreover, AP-1 protein activities are tightly regulated by a series of upstream kinases such as MAPK cascades through modifications [5]. Recently, specific AP-1 dimers have been shown to be associated with carcinogenesis and cancer development. For example, the c-Jun/c-Fos dimer was shown to promote proliferation in lung cancer via upregulating cyclin D1 [6]. Fra-1 was shown to associate with c-Jun, transactivating MMP-1 to increase malignant invasion in breast cancer [7] and osteosarcoma cells [8]. In glioma cells, Fra-1 was found to upregulate and dimerize with JunB, contributing to the malignant phenotype [9]. Thus, identifying and inactivating specific AP-1 dimers with oncogenic roles appear to be a promising strategy for impairing malignant phenotypes in different tumor types.

Histone deacetyltransferases (HDACs) were originally identified as a family of enzymes that remove acetyl groups from histones to balance the process of histone acetyltransferases (HAT)-mediated histone acetylation, which is critical for regulating gene transcription. Recently, abnormal HDAC activities have been reported to be associated with a number of human cancers, and HDAC inhibitors (HDACIs) have been developed as a novel therapeutic class of drugs for treating different types of tumors [10]. Based on their chemical structures, four types of HDACIs have been characterized, including short-chain fatty acids (sodium butyrate, valproic acid (VPA), a clinical drug for treating epilepsy), hydroxamic acids (suberoylanilidehydroxamic acid (SAHA), trichostatin A (TSA) and LBH589), synthetic benzamide derivatives (MS-275, M-344) and cyclic tetrapeptides (depsipeptide), and SAHA has been proved by Food and Drug Administration (FDA) to treat cutaneous T-cell lymphoma (CTCL) in clinic. Treatment of tumor cell lines with HDACIs commonly induces cell cycle arrest, which is associated with the transcriptional alteration of many genes critical for the cell cycle, such as p21WAF1/CIP1 [11] and cyclins A and D [12]. Moreover, HDACIs can induce apoptosis of cancer cells through activation of proapoptotic BH3-only proteins (i.e., Bid, Bim) [13, 14], stimulation of JNK activity [15] or stabilization of p53 [16]. In addition, HDACIs can suppress the migration and invasion of tumor cells by downregulating MMP-2 and MMP-9 expression [17, 18]. Thus, HDACIs have emerged as a powerful new class of small-molecular therapeutics with a wide range of anticancer activities.

In this study, we attempted to clarify an AP-1 dimer that is critical for proliferation in NB cells and to determine whether HDACIs can suppress oncogenic AP-1 dimers. Using the NB cell lines with MYCN single copy or MYCN amplified, we identified that Fra-1 dimerization with c-Jun promotes NB cell proliferation. Furthermore, we found HDACIs suppress the dimer via transcriptionally downregulating MKK7 and Raf1. Our findings provided new insight into the molecular mechanism of tumor suppression for HDACIs in selectively targeting oncogenic AP-1 dimers and their upstream cascades.

## RESULTS

### HDACIs transcriptionally suppressed both c-Jun and Fra-1, preceding the inhibitory effects on cell proliferation

**Figure 1:**
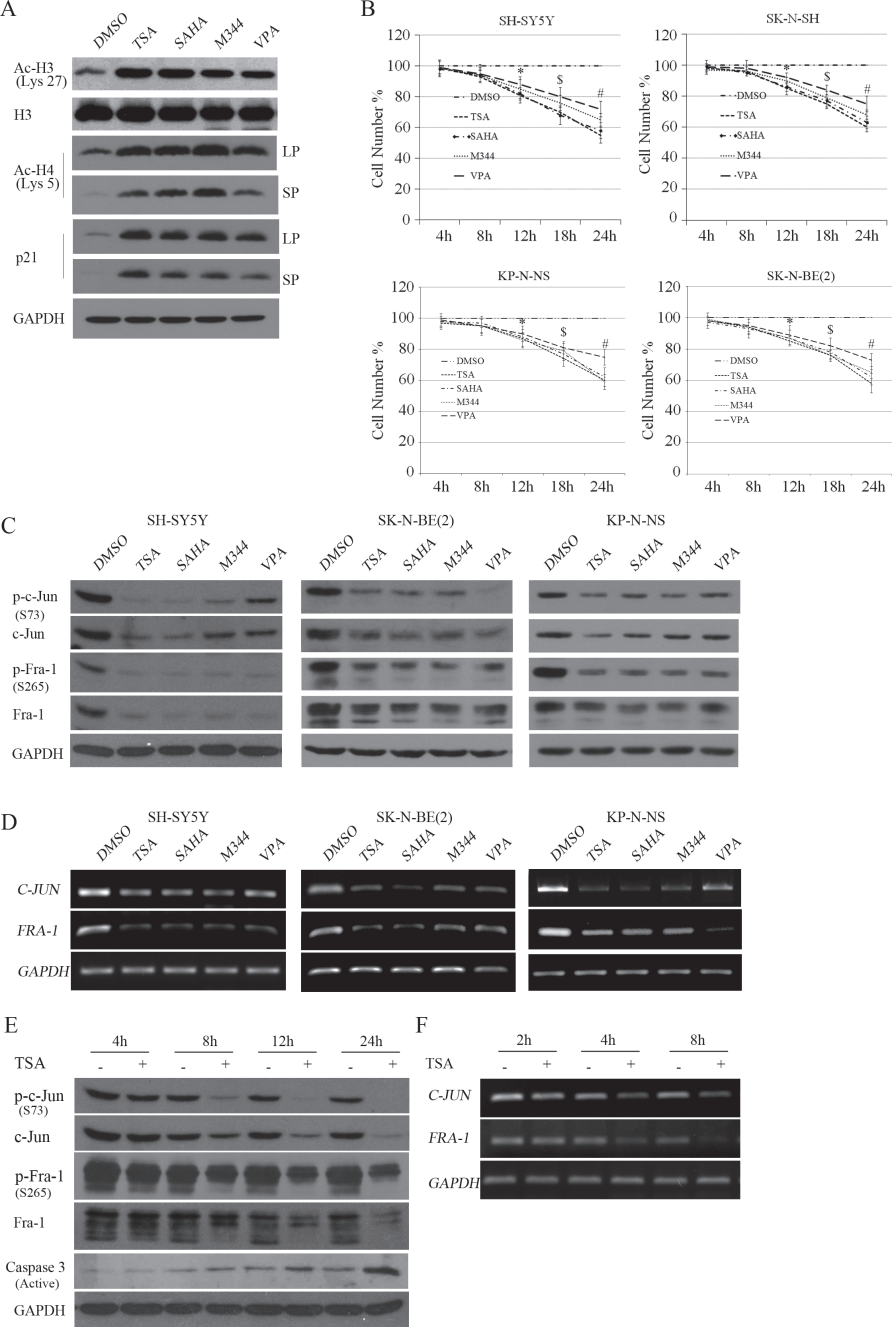
HDACI-induced transcriptional suppression of c-Jun and Fra-1 occurs before the inhibitory effects on cell proliferation (**A**) SH-SY5Y cells were treated with HDACIs, including 0.5 μM TSA, 1 μM SAHA, 2 mM VPA or 1 μM M344 for 2 hours, and WB was performed to test H3, H3 K27 and H4 K5 acetylation, p21, GAPDH was reprobed to verify equal loading. LP: Long time of exposure; SP: Short time of exposure. (**B**) SH-SY5Y, SK-N-SH, SK-N-BE(2), KP-N-NS cells were treated with HDACIs for 4, 8, 12, 18, and 24 hours, and MTT assays were performed to determine the proliferation rates at each time point. The data are presented as the mean ± S.E. *n* = 3; Two-way ANOVA analysis, *P < 0.05 (Control vs. HDACI at 12 hours), $*P* < 0.05 (HDACI at 12 hours vs. HDACI at 18 hours), #*P* < 0.05 (HDACI at 18 hoursvs. HDACI at 24 hours). (**C** and **D**) SH-SY5Y, SK-N-BE(2) and KP-N-NS cells were treated with the four HDACIs for 12 hours, and WB was performed to detect the expression and phosphorylation of c-Jun and Fra-1. GAPDH was reprobed to verify equal loading. RT-PCR was performed to detect the mRNA levels of c-Jun and Fra-1. GAPDH was amplified as an equal input. (**E** and **F**) SH-SY5Y cells were treated with 0.5 μM TSA for 4, 8, 12 and 24 hours, and WB was performed to detect the expression and phosphorylation of c-Jun and Fra-1. RT-PCR was performed to detect the mRNA levels of c-Jun and Fra-1.

HDACIs can effectively inhibit cell viability at low concentrations, and high concentrations of HDACIs commonly evoke additional pathways, leading to strong cell growth arrest and ultimately resulting in apoptosis. We treated two NB cell lines with single MYCN copy, SK-N-SH and SH-SY5Y, and two NB cells with MYCN amplified, SK-N-BE(2) and KP-N-NS, with three types of broad-spectrum HDAC inhibitors, including short-chain fatty acids (VPA), hydroxamic acids (TSA and SAHA), and synthetic benzamide derivatives (M344), and observed the dynamic correlation between cell viability and the time course of HDACI treatment. Treating cells with 500 nM TSA, 1 μM SAHA, 1 μM M344 or 2 mM VPA for 1 hour induced a robust increase in both H3 lysine 27 and H4 lysine 12 acetylation (Figure 1A), and also remarkably induces p21 expression [12], suggesting that HDAC activities were efficiently suppressed by the HDACIs used. MTT assays demonstrated that all HDACIs used remarkably inhibited the proliferation of the four cell lines starting at 12 hours post-treatment and induced greater suppression lasting up to 18 and 24 hours (*P* < 0.05, Figure 1B). These results suggested that HDACI treatment substantially reduced cellular viability and proliferation in NB cells, consistent with previous reports [19, 20].

c-Jun has been shown to be an oncogene or tumor suppressor, largely depending on the cell type or stress condition [21]. Thus, we detected whether c-Jun was altered following HDACI treatment in NB cells. Interestingly, SH-SY5Y, SK-N-BE(2) and KP-N-NS cells subjected to HDACIs for 12 hours exhibited dramatic decreases in c-Jun expression and phosphorylation (the activated form) levels. Paralleling the decreased c-Jun expression, HDACI treatment also induced decreases in Fra-1 expression and phosphorylation (activated form) levels (Figure 1C). RT-PCR assays demonstrated that both c-Jun and Fra-1 mRNA levels were transcriptionally downregulated by HDACIs (Figure 1D). Notably, the four HDACIs exhibited different inhibitive effects on c-Jun or Fra-1, probably due to their variable sensitivity and specificity in blocking the activity of the HDAC member(s) critical for sustaining c-Jun or Fra-1 expression. To observe the time course of the inhibitory effects of HDACIs on c-Jun and Fra-1 expression, we used 500 nM TSA to treat cells for different time durations (4, 8, 12, and 24 hours). As shown in Figure 1E, TSA treatment led to obvious decreases in c-Jun and Fra-1 phosphorylation and protein levels starting at 8 hours and lasting up to 12 hours. At 24 hours post-treatment, when typical apoptosis occurred with active caspase 3, c-Jun and Fra-1 remained suppressed by TSA treatment. c-Jun and Fra-1 mRNA expression levels were suppressed before the decrease in their protein expression levels starting at 4 hours and lasting up to 8 hours (Figure 1F). In SK-N-SH cells, HDACI also consistently led to the downregulation of c-Jun and Fra-1 protein and mRNA levels (Supplementary Data S1; Figure 1). Taken together, these results indicated that HDACIs caused the transcriptional downregulation of both c-Jun and Fra-1, preceding their inhibitory effect on cell proliferation.

### c-Jun dimerization with Fra-1 predominantly occupied the TRE site responsible for TRE activity

**Figure 2:**
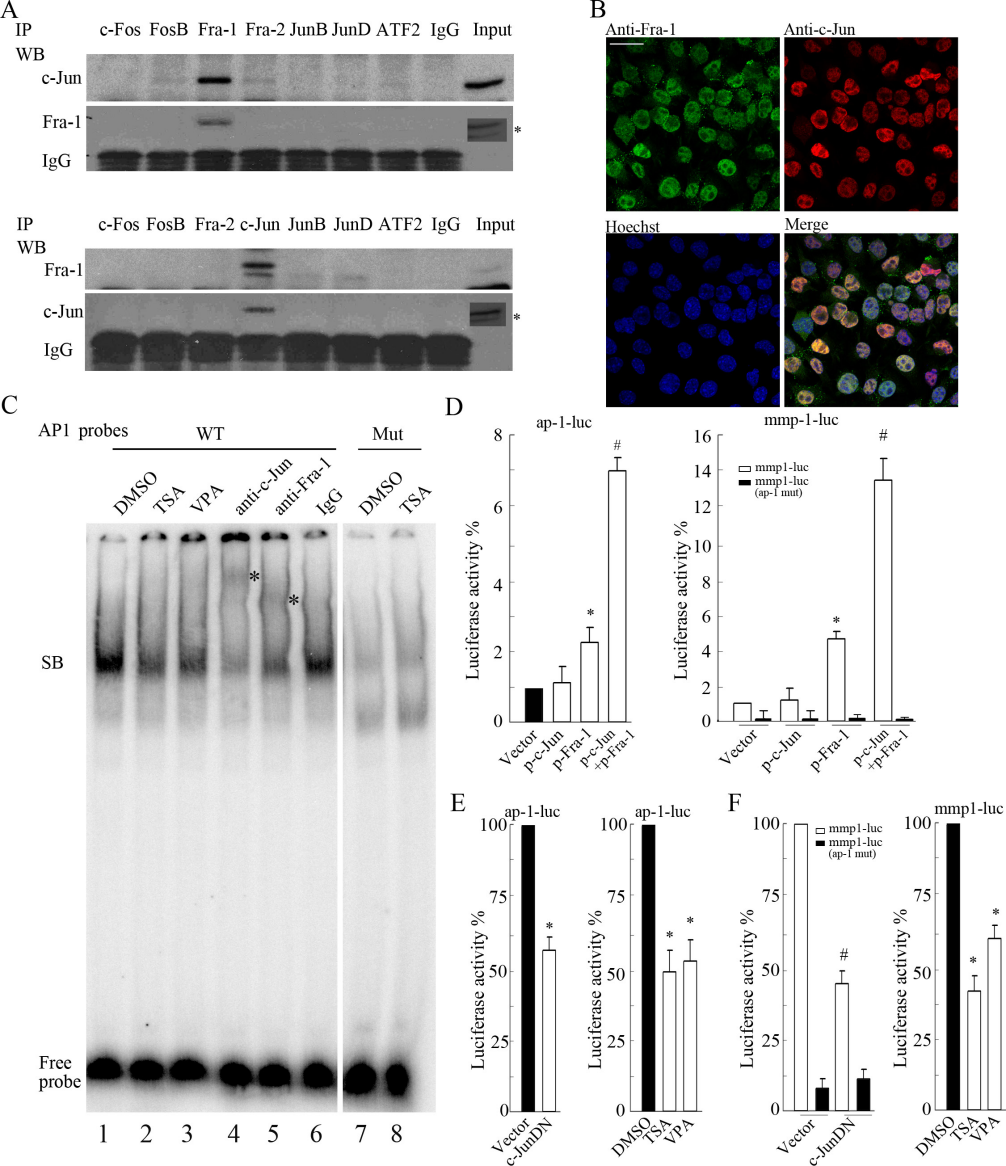
c-Jun primarily interacting with Fra-1 as a dimer contributes to TRE activity (**A**) SH-SY5Y cells were lysed for IP assays, and c-Jun or Fra-1 was detected by WB in the precipitates pulled down by antibody against c-Fos, FosB, Fra-1, Fra2, c-Jun, JunB, JunD, ATF2 or normal IgG. Input indicates the cellular lysate before adding antibodies. *Signal from long time of exposure. (**B**) IF with double staining was performed to detect c-Jun and Fra-1 expression in SH-SY5Y cells. Nuclei were stained with Hoechst 33258. All images were acquired in the same field by confocal microscope (scale bar = 10 μm). (**C**) A gel mobility shift assay was performed using a ^32^P-labeled wild type (WT) AP-1 probe or mutant (Mut) AP-1 probe with nuclear extracts prepared from SH-SY5Y cells. Antibodies against c-Jun and Fra-1 were pre-incubated with nuclear extracts to detect the composition of DNA-protein complexes, and normal IgG was used as an antibody control. SB: the specific DNA-protein band. (**D** and **E**) SH-SY5Y cells were transfected with ap-1-luc, mmp1-luc or mmp1-luc mutant (ap-1 mut) plasmids and pCMV-RL in combination with or without the plasmids encoding c-JunDN. Twenty-four hours after transfection, cells were subjected to dual reporter analysis directly or after 12 hours of TSA or VPA treatment. Luciferase activity was normalized to RL activity. The data are presented as the mean ± S.E. *n* = 3; One-way ANOVA with selected pairs analysis, #*P* < 0.05 (Vector vs. c-JunDN), *P < 0.05 (DMSO vs. HDACI).

To clarify the major dimerization partner for c-Jun or Fra-1 in SH-SY5Y cells, cell lysates were immunoprecipitated with antibodies against AP-1 members that have been shown to be able to interact with c-Jun or Fra-1 to form homo-/heterodimers. These AP-1 members include c-Fos, FosB, Fra-1, Fra2, c-Jun, JunB, JunD and ATF2. The precipitates were analyzed by WB with c-Jun or Fra-1 monoclonal antibody. All antibodies used against the above AP-1 members worked well in precipitating the respective antigens (data not shown). Interestingly, c-Jun was detected primarily in the precipitates pulled down by Fra-1 antibody but not in those pulled down by c-Fos, Fos B, Fra2, JunB, JunD or ATF2 antibody (Figure 2A). Consistently, Fra-1 was only present in the c-Jun antibody-immunoprecipitated complexes and was not detected in other pulled down complexes. Further analysis by ICC indicated that c-Jun and Fra-1 physically co-localized to the nucleus (Figure 2B). Co-IP results showed that c-Jun and Fra-1 also forms a dimer in SK-N-SH, SK-N-BE(2) and KP-N-NS cell lines (Supplementary Data S2; Figure 2).

Next, we performed a gel mobility shift assay to determine whether c-Jun/Fra-1 heterodimers exist in the nucleus and bind to the conserved TRE site using an end-labeled AP-1 probe containing the 7 bp TRE sequence (TGACTCA). Indeed, the AP-1 probe could capture a complex, whereas the mutated AP-1 probes could not (Figure 2C, lanes 1, 7, and 8). HDACIs including TSA and VPA induced a marked decrease in the intensity of the complex due to the suppression of c-Jun and Fra-1 expression (Figure 2C, lanes 2 and 3). The addition of c-Jun or Fra-1 antibody led to a light and smeared super-shift band, in contrast to the reduced specific complex compared with the addition of normal IgG (Figure 2B, lanes 4, 5, and 6). No appearance of clear super-shifted band is probably due to the effect that addition of antibody brings an obstacle to labeled probes binding to the AP-1 complex. These results indicated that both c-Jun and Fra-1 were present in the complex. The trace amounts of complex did not super-shift after the addition of excess c-Jun and Fra-1 antibodies, suggesting that other factors not yet determined might exist in the complex captured by the probes.

To determine whether c-Jun/Fra-1 heterodimerization contributes to the activation of promoters containing the TRE element (TGACTCA), we conducted reporter gene assays using 7 × AP-1-luciferase (ap-1-luc) and mmp1-luciferase (mmp1-luc) reporters. As shown in Figure 2D and 2E, both reporters containing the conserved TRE site (TGACTCA) displayed high activity levels in SH- SY5Y cells; the mmp-1-luc mutant with a mutated TRE site displayed strongly reduced activity. c-JunDN, which lacked the transactivation domain but that could bind to Fra-1 or c-Jun to block their function, greatly abrogated ap-1-luc and mmp1-luc activities (*P* < 0.05). Moreover, HDACIs remarkably inhibited the activities of the reporters due to their inhibition of both c-Jun and Fra-1 expression (*P* < 0.05). Taken together, these results indicated that c-Jun heterodimerization with Fra-1 binds to the conserved TRE site responsible for TRE activity in NB cells.

### c-Jun/Fra-1 heterodimerization is essential for NB cell proliferation

**Figure 3:**
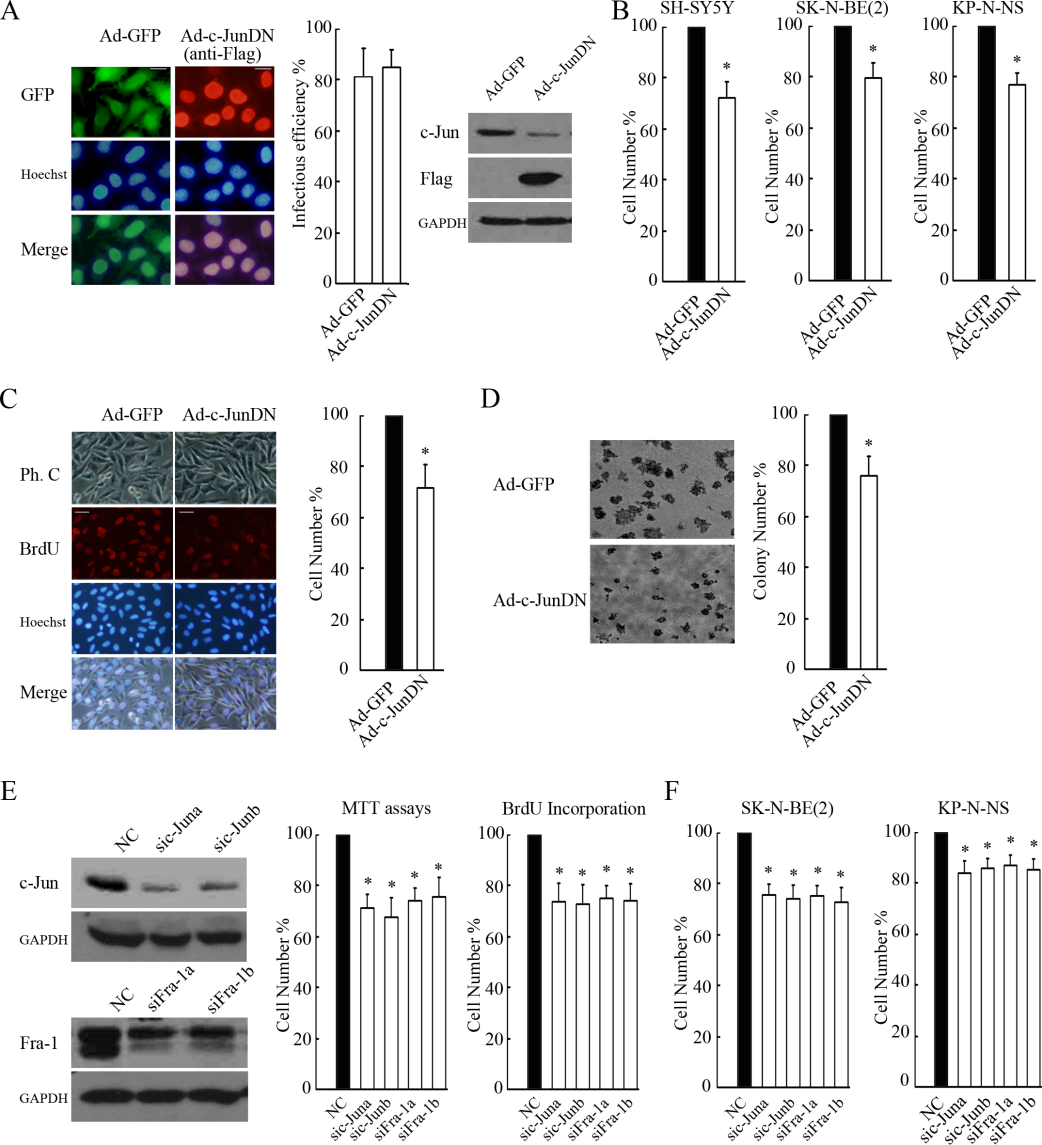
c-Jun/Fra-1 dimer-mediated TRE activity is essential for cell proliferation (**A**) SH-SY5Y cells were infected with 100 MOI Ad-c-JunDN or control Ad-GFP for 48 hours, and IF was performed to determine infectious efficiency by calculating GFP or Flag-positive cells/total cells × 100% (scale bar = 10 μm). WB was performed to determine the function of c-JunDN expression in the suppression of c-Jun. (**B** and **C**) SH-SY5Y, SK-N-BE(2) and KP-N-NS cells were infected with 100 MOI Ad-c-JunDN or control Ad-GFP for 48 hours, and MTT assays or BrdU incorporation were performed to determine proliferation rates (scale bar = 20 μm). (**D**) SH-SY5Y cells infected with Ad-c-JunDN or Ad-GFP (MOI 100) were seeded onto plates at 1 × 10^3^ cells/ml for colony formation assays. The number of colonies with a diameter > 50 μm was counted under a microscope, and images were acquired at 40× magnification. The data are presented as the mean ± S.E. *n* = 3; One-way ANOVA with selected pairs analysis, *P < 0.05 (Ad-GFP vs. Ad-c-JunDN). (**E** and **F**) SH-SY5Y, SK-N-BE(2) and KP-N-NS cells were transfected with non-specific siRNA control (NC), sic-Juna, sic-Junb, siFra-1a or siFra-1b for 48 hours, and WB was performed to detect their respective silencing efficiency on c-Jun and Fra-1 expression. MTT assays or BrdU incorporation were performed to determine proliferation rates. The data are presented as the mean ± S.E. *n* = 3; One-way ANOVA with selected pairs analysis, *P < 0.05 (NC vs. siRNA).

To examine whether c-Jun/Fra-1 heterodimer-mediated TRE activity was essential for NB proliferation, we used c-JunDN to block c-Jun and Fra-1 functions. Adenovirus-delivered c-JunDN was expressed in most cells and successfully inhibited c-Jun expression because c-Jun is a self-regulated gene (Figure 3A). Expression of c-JunDN in SH-SY5Y, SK-N-BE(2), KP-N-NS significantly decreased proliferation rates by 28 ± 7%, 21 ± 6%, 23 ± 4% compared with the control as determined by MTT assays, respectively (*P* < 0.05, Figure 3B). Consistently, c-JunDN also induced an obvious decrease in the number of BrdU-positive cells compared with the control (*P* < 0.05, Figure 3C). Moreover, the abrogation of c-Jun/Fra-1 function by c-JunDN suppressed the proliferation and malignant transformation of cells as determined by colony formation assays (*P* < 0.05, Figure 3D). Because c-JunDN might affect the activity of other AP-1 transcription factors, we introduced siRNA targeting c-Jun or Fra-1. Accordingly, c-Jun or Fra-1 knockdown effectively reduced cellular growth compared with the control (*P* < 0.05, Figure 3E). The similar inhibition on proliferation could be observed when applying c-JunDN, knockdown of c-Jun or Fra-1 to SK-N-SH, SK-N-BE(2) and KP-N-NS cells (Figure 3F and Supplementary Data S2). These results demonstrated that c-Jun/Fra-1 heterodimer-mediated TRE activity was essential for NB cell proliferation.

### Co-overexpression of c-Jun and Fra-1 promoted proliferation and antagonized HDACI-mediated inhibitory effects

**Figure 4:**
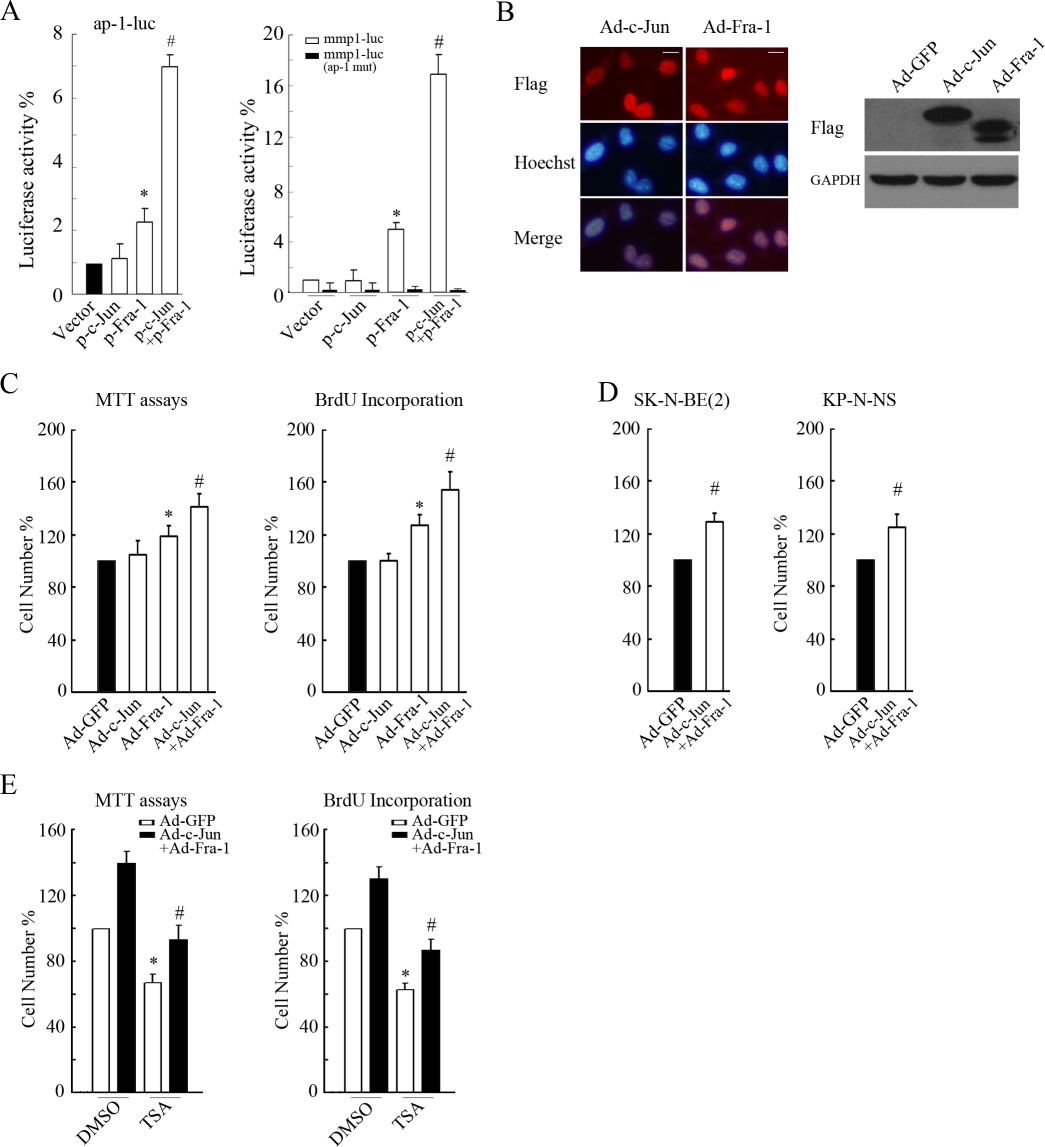
Co-overexpression of c-Jun and Fra-1 promoted proliferation and antagonized HDACI-mediated inhibitory effects (**A**) SH-SY5Y cells transfected with ap-1-luc, mmp1-luc or mmp1-luc mutant (ap-1 mut) plasmids and pCMV-RL in combination with or without the plasmids expressing c-Jun or Fra-1 alone or co-expressing c-Jun+Fra-1 were subjected to dual reporter analysis 24 hours after transfection. The data are presented as the mean ± S.E. *n* = 3; One-way ANOVA with selected pairs analysis, *P < 0.05 (Vector vs. Fra-1), #*P* < 0.05 (Vector vs. c-Jun + Fra-1). (**B**) SH-SY5Y cells were infected with 100 MOI Ad-c-Jun or Ad-Fra-1 for 48 hours, and IF or WB was performed to determine their infectious efficiency or to clarify their expression, respectively (scale bar = 10 μm). (**C** and **D**) SH-SY5Y, SK-N-BE(2) and KP-N-NS cells were infected with 100 MOI Ad-c-Jun or Ad-Fra-1 alone or Ad-c-Jun + Ad-Fra-1. Ad-GFP was included as control. Forty-eight hours later, MTT assays or BrdU incorporation were performed to determine proliferation rates. The data are presented as the mean ± S.E. *n* = 3; One-way ANOVA with selected pairs analysis, *P < 0.05 (Ad-Fra-1 vs. Ad-GFP), #*P* < 0.05 (Ad-c-Jun + Ad-Fra-1 vs. Ad- Fra-1). (**E**) SH-SY5Y cells were infected with Ad-c-Jun + Ad-Fra-1, and Ad-GFP was included as control. Forty-eight hours later, infected cells were treated with 0.5 μM TSA for 24 hours, and MTT assays or BrdU incorporation were performed to determine proliferation rates. The data are presented as the mean ± S.E. *n* = 3; One-way ANOVA with selected pairs analysis, *P < 0.05 (DMSO: Ad-GFP vs. Ad-c-Jun + Ad-Fra-1); #*P* < 0.05 (TSA: Ad-GFP vs. Ad-c-Jun + Ad-Fra-1).

To further examine the role of c-Jun/Fra-1 dimers in regulating NB cell growth, we investigated the effects of co-ectopic c-Jun and Fra-1 expression on TRE activity and proliferation in NB cells. Dual reporter assays showed that compared with the control, ectopic expression of c-Jun alone did not increase ap-1-luc or mmp-1-luc activity levels, overexpression of Fra-1 only slightly increased their activity levels to approximately 2.2- and 5.0-fold, respectively, but co-expression of c-Jun and Fra-1 greatly augmented the activity levels of the two reporters to approximately 7.1- and 17.0-fold, respectively (Figure 4A). Similar to the response of TRE activity to ectopic c-Jun and Fra-1, adenovirus-mediated overexpression of c-Jun had no obvious influence on cellular proliferation (*P* > 0.05), and overexpression of Fra-1 significantly promoted proliferation compared with the control (*P* < 0.05). Co-expression of c-Jun and Fra-1 induced a greater number of cells to propagate in the two MYCN single copy cells, as well as in the MYCN amplified NB cells (*P* < 0.05, Figure 4B, 4C, 4D and Supplementary Data S2). These results indicated that enhanced c-Jun and Fra-1 activity synergistically promoted cellular growth. As expected, co-overexpression of c-Jun and Fra- 1 remarkably relieved TSA-induced growth inhibition compared with the control (*P* < 0.05, Figure 4E, Supplementary Data S2), suggesting that the elimination of c-Jun and Fra-1 expression was involved in the inhibitory effects of HDACIs on proliferation.

Taken together, these results indicated that c-Jun/Fra-1 heterodimer-mediated TRE activity was critical for the proliferation of NB cells.

### HDACIs suppressed MEK/ERK-mediated Fra-1 expression through transcriptionally downregulating Raf1

**Figure 5:**
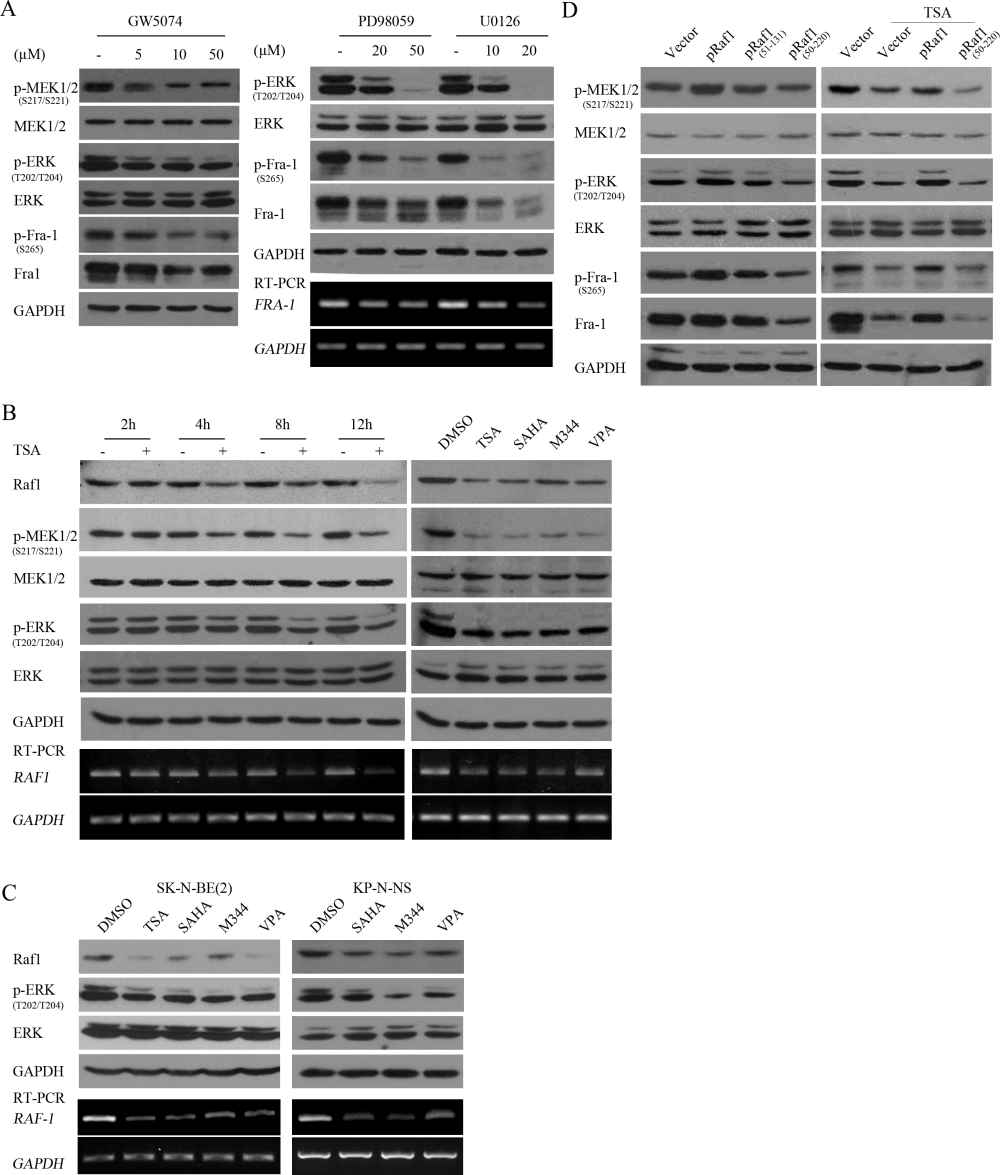
HDACIs suppressed Fra-1 expression through transcriptionally downregulating Raf1 and consequently decreasing MEK1/2-ERK1/2 activity (**A**) SH-SY5Y cells treated with GW5074, PD98059 or U0126 at different doses for 4 hours were subjected to WB with antibody against phosphorylated MEK1/2, MEK1/2, p-ERK, ERK, phosphorylated Fra-1 or Fra-1; GAPDH was reprobed to verify equal loading. Total mRNA was extracted and subjected to RT-PCR with specific primers against Fra-1, and GAPDH was amplified to verify equal input. (**B**) SH-SY5Y cells treated with 0.5 μM TSA for different time courses (right panel), or the 4 HDACIs (left panel) were lysed and subjected to WB with antibody against Raf1, phosphorylated MEK1/2, MEK1/2, phosphorylated ERK, ERK. GAPDH was reprobed to verify equal loading. RT-PCR was performed with specific primers against Raf1, and GAPDH was amplified to verify equal input. (**C**) SK-N-BE(2) and KP-N-NS cells treated with the 4 HDACIs were lysed and subjected to WB with antibody against Raf1, phosphorylated ERK, ERK. GAPDH was reprobed to verify equal loading. RT-PCR was performed with specific primers against Raf1, and GAPDH was amplified to verify equal input. (**D**) SH-SY5Y cells transfected with control vector pcDNA 3.1, plasmids expressing Raf1, dominant negative mutant Raf1 (51–131) or Raf1 (51–220) were treated without (right panel) or with 0.5 μM TSA treatment for 12 hours (left panel), and then WB was performed with antibody against Raf1, phosphorylated MEK1/2, MEK1/2, phosphorylated ERK, ERK, phosphorylated Fra-1 or Fra-1. GAPDH was reprobed to verify equal loading.

Fra-1 accumulation has been shown to critically depend on Raf-MEK1/2-ERK1/2 activity-dependent transcriptional regulation and posttranslational stabilization [22, 23]. Indeed, GW5074, a specific inhibitor of Raf1, dose-dependently inhibited MEK1/2 and ERK1/2 activities and Fra-1 expression (Figure 5A). Inhibition of the MEK1/2-ERK1/2 pathway by U0126 or PD98059 consistently abrogated Fra-1 mRNA expression levels and protein phosphorylation and expression levels in SH- SY5Y cells. As expected and consistent with previous reports [24], blocking MEK1/2-ERK1/2 activity efficiently inhibited cell growth (data not shown).

To determine whether HDACI-mediated suppression of Fra-1 was due to inhibition of Raf1-MEK1/2-ERK1/2 activity, dynamic changes in Raf1 expression, MEK1/2-ERK1/2 activities and Fra-1 expression were analyzed following TSA treatment for different time courses. TSA induced an obvious reduction in MEK1/2 and ERK1/2 activities at 4 hours post-treatment in SH-SY5Y and SK-N-SH cells, which is before the decrease in Fra-1 caused by TSA. In contrast, TSA mediated the downregulation of Raf1 mRNA and protein at 2 hours post-treatment, earlier than the decrease in MEK1/2 activity (Figure 5B, Supplementary Data S2). Administration of all HDACIs used transcriptionally downregulated Raf1 expression and decreased MEK1/2 and ERK1/2 activities in SH-SY5Y, SK-N-BE (2) and KP-N-NS (Figure 5B, 5C). Furthermore, Raf1 overexpression increased MEK1/2-ERK1/2 activity and efficiently rescued the HDACI-mediated suppression of Fra-1, whereas dominant negative mutant Raf1 (51–220) inhibited MEK1/2-ERK1/2 activity and Fra-1 expression and did not rescue HDACI-mediated Fra-1 suppression (Figure 5D and Supplementary Data S2). These results suggested that HDACIs targeted Fra-1 expression through transcriptionally downregulating Raf1 and consequently decreasing MEK1/2-ERK1/2 activity.

### MKK7, but not MKK4, mediated JNK/c-Jun activation and proliferation in NB cells

**Figure 6:**
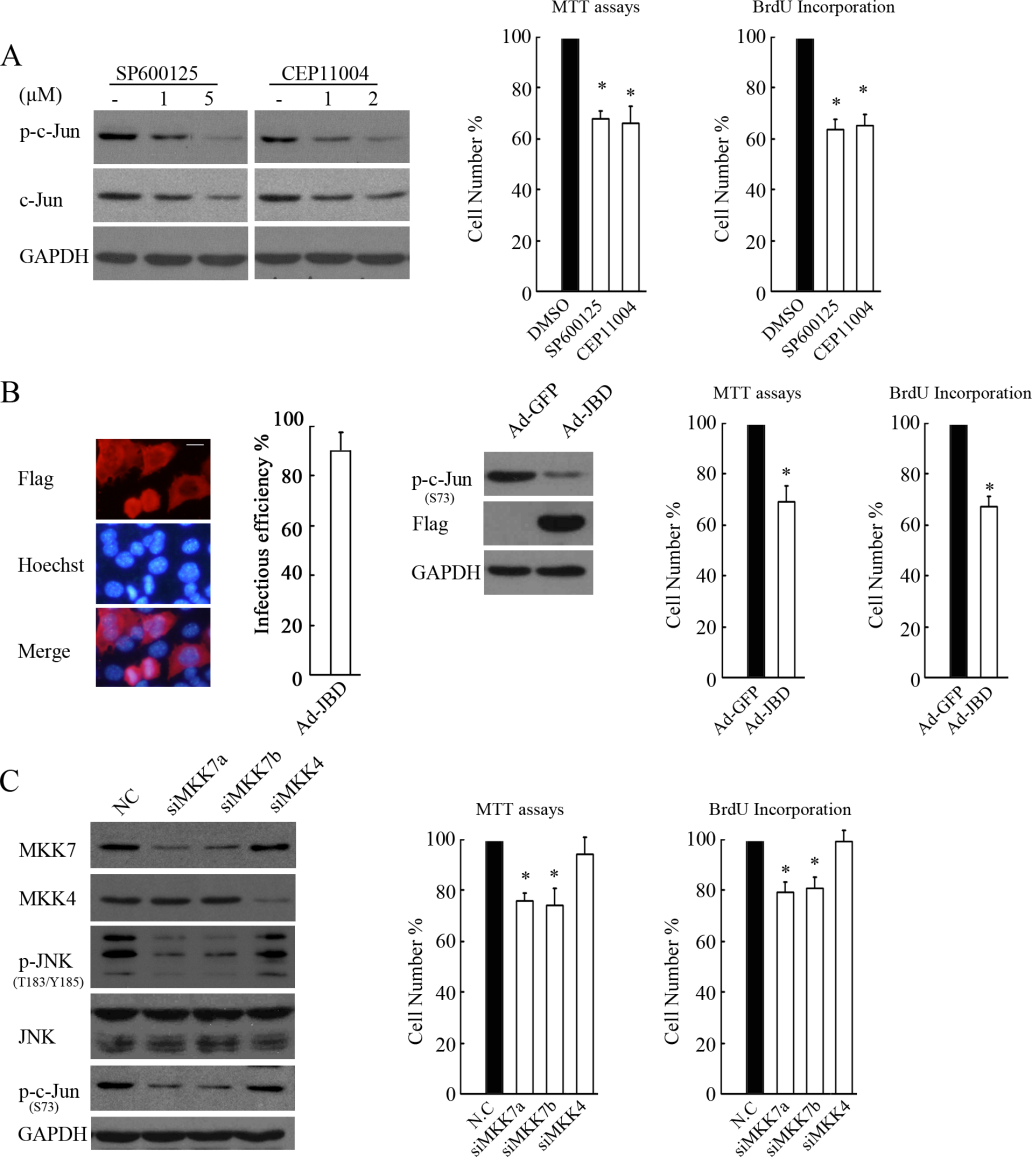
MKK7, but not MKK4, mediates JNK/c-Jun activation and proliferation in SH-SY5Ycells (**A**) SH-SY5Y cells treated with SP600125 or CEP11004 at different doses for 4 hours were subjected to WB with antibody against phosphorylated c-Jun or c-Jun. GAPDH was reprobed to verify equal loading. SH-SY5Y cells treated with 5 μM SP600125 or 2 μM CEP11004 for 24 hours were subjected to MTT assays or BrdU incorporation to determine proliferation rates. The data are presented as the mean ± S.E. *n* = 3; One-way ANOVA with selected pairs analysis, *P < 0.05 (DMSO vs. Inhibitor). (**B**) SH-SY5Y cells were infected with 100 MOI Ad-JBD or control Ad-GFP for 48 hours, and then IF or WB was performed to determine the infectious efficiency or the function of expressing JBD in suppression of c-Jun, respectively (scale bar = 10 μm). MTT assays or BrdU incorporation was performed to determine the proliferation rate. The data arepresented as the means ± S.E., *n* = 3; One-way ANOVA with selected pairs analysis, *P < 0.05 (Ad-GFP vs. Ad-JBD). (**C**) SH-SY5Y cells were transfected with non-specific siRNA control (NC), siMKK7a, siMKK7b, or siMKK4 for 48 hours, and then WB was performed with antibody against MKK7, MKK4, phosphorylated JNK, JNK, and c-Jun. MTT assays and BrdU incorporation were performed to determine proliferation rates. The data are presented as the mean ± S.E. *n* = 3; One-way ANOVA with selected pairs analysis, *P < 0.05 (NC vs. siRNA).

MKK4 and MKK7 have been shown to activate JNK by preferentially phosphorylating JNK on Tyr 183 and Thr 185. MKK7 is commonly essential for stress-stimulated JNK activation, whereas MKK4 deficiency causes a reduction in basal JNK activity [25]. Thus, we determined whether MKK4 or MKK7 was essential for basal JNK activation and cellular proliferation in SH-SY5Y cells. Administration of the JNK inhibitor SP600125 or MLK3 (an upstream kinase of MKK7 or MKK4) inhibitor CEP11004 greatly abrogated c-Jun and proliferation, suggesting that the MLK3-MKK4 or 7-JNK cascade contributes to c-Jun activation and cell viability and proliferation (Figure 6A). JNK interacting protein (JIP)-1 is a scaffold protein that interacts with JNK and its upstream activating kinases MLK3 and MKK7 but not MKK4, and its JBD (residues 127–281) exhibits a high affinity to JNK and can thus specifically block JNK from being activated by MLK3-MKK7 [26]. Indeed, adenovirus-mediated JBD expression suppressed c-Jun activity and cell proliferation (Figure 6B). Furthermore, MKK7 knockdown by siRNAs consistently reduced JNK/c-Jun activation and cell proliferation, whereas silencing MKK4 had no such effects (Figure 6C). These results clearly demonstrated that MKK7, not MKK4, contributes to JNK/c-Jun activation and proliferation in SH-SY5Y cells.

### HDACIs suppressed JNK activity through transcriptionally downregulating MKK7

**Figure 7:**
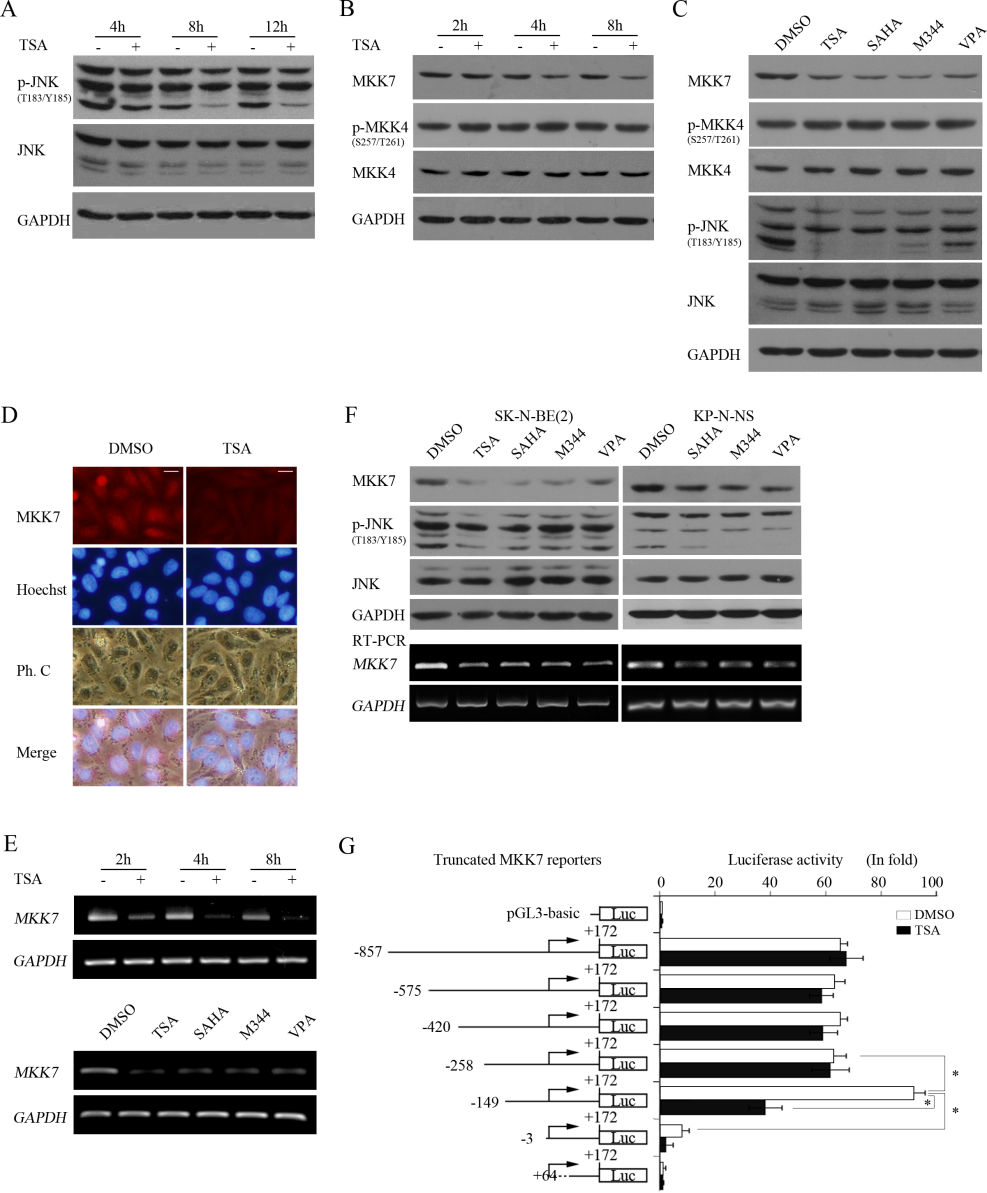
HDACIs transcriptionally downregulate MKK7 and consequently suppress JNK activity (**A** and **B**) SH-SY5Y cells treated with 0.5 μM TSA for different time course were lysed and subjected to WB with antibody against phosphorylated JNK and JNK (A) or MKK7, phosphorylated MKK4, and MKK4, respectively. GAPDH was reprobed to verify equal loading. (**C**) SH-SY5Y treated with the 4 HDACIs for 8 hours were subjected to WB with antibody against MKK7, phosphorylated MKK4, MKK4, phosphorylated JNK, and JNK. GAPDH was reprobed to verify equal loading. (**D**) SH-SY5Y cells were treated with 0.5 μM TSA for 8 hours, and then IF was performed to detect MKK7 expression (scale bar = 10 μm). (**E**) Cells were treated with 0.5 μM TSA for 2, 4, 8 hours, and total mRNA was extracted and subjected to RT-PCR with specific primers for MKK7. GAPDH was amplified to verify equal input. (**F**) SK-N-BE(2) and KP-N-NS cells treated with the 4 HDACIs were lysed and subjected to WB with antibody against MKK7, phosphorylated JNK, and JNK. GAPDH was reprobed to verify equal loading. RT-PCR was performed with specific primers against MKK7, and GAPDH was amplified to verify equal input. (**G**) Structures of *MKK7* promoter 5′ sequential deletion constructs are shown. *MKK7* promoter fragments of different lengths but with the same 3′-end were cloned into pGL3-Basic. SH-SY5Y cells transfected with reporter plasmids and pCMV-RL for 12 h were subjected to DMSO or TSA treatment for 12 hours, and then dual reporter analysis was performed. The data are presented as the mean ± S.E. *n* = 3; One-way ANOVA with selected pairs analysis, *P < 0.05.

To explore the mechanisms involved in HDACI-mediated c-Jun suppression, we investigated whether HDACIs could inhibit JNK activity. Using TSA to treat cells for different durations (4, 8 and 12 hours), we found that JNK phosphorylation was remarkably decreased at 8 hours post-TSA treatment (Figure 7A and Supplementary Data S2), concomitant with the suppression of c-Jun expression induced by HDACIs. Lasting TSA treatment resulted in continued suppression of JNK activity but without protein level changes, suggesting that the target of TSA is upstream of JNK.

We then examined whether TSA treatment decreased MKK7 expression. Interestingly, TSA treatment downregulated MKK7 at 4 hours post-treatment to 8 hours, which is earlier than the decrease in JNK activity, but did not alter MKK4 expression and phosphorylation (Figure 7B and Supplementary Data S2). All HDACIs decreased MKK7 expression without altering MKK4 phosphorylation or expression (Figure 7C). Furthermore, IF analysis consistently indicated that MKK7 expression was reduced upon TSA treatment (Figure 7D). These results indicated that HDACI treatment substantially inhibited MKK7 expression.

Because HDACI treatment commonly alters gene expression in transcription, we assessed whether MKK7 mRNA levelswere accordingly downregulated by HDACIs, leading to protein expression changes. RT-PCR indicated that MKK7 mRNA levels were decreased starting at 2 hours post-TSA treatment and decreased further with continued TSA treatment. Moreover, all HDACIs effectively downregulated MKK7 mRNA expression (Figure 7E). In MYCN amplified cells, HDACIs also inhibited MKK7 expression in protein and mRNA levels (Figure 7F). These results indicated that HDACI treatment caused transcriptional suppression of MKK7.

To further support the finding that HDACIs transcriptionally inhibit MKK7, we constructed a reporter gene containing a DNA fragment spanning from −857 to +172 of the *MKK7* 5′-flanking region relative to the transcription start site and detected its basic activity and HDACI treatment response. The 1029 bp reporter exhibited 60-fold higher luciferase activity than pGL3-Basic. However, the reporter did not exhibit a significant response to TSA compared to the control (Figure 7G).

To define the critical sequence responsible for *MKK7* promoter activation and response to HDACI treatment, we constructed a series of truncated reporters based on the original *MKK7* (−857/+172)-luc containing various lengths of the *MKK7* 5′-flanking regions but with a common 3′-end. Dual reporter assays indicated that the truncated reporter *MKK7* (−575/+172)-luc, *MKK7* (−420/+172)-luc or *MKK7* (−258/+172)-luc was as active as the original construct but still had no response to TSA treatment. However, when the fragment was deleted to −149, *MKK7* (−149/+172)-luc activity increased by approximately 2-fold compared with that of *MKK7* (−258/+172)-luc and approximately 100-fold than that of pGL3-Basic. More interestingly, the reporter exhibited a strong response to HDACI treatment, and its activity was sharply decreased by 60% upon TSA treatment, consistent with the response of MKK7 mRNA and protein to HDACIs. With fragment deletion to −3, *MKK7* (−3/+172)-luc activity was greatly decreased by approximately 93% compared to that of *MKK7* (−149/+ 172)-luc (Figure 7G). When the fragment was deleted to + 68, *MKK7* (+ 68/+ 172)-luc activity was completely abrogated, and the TSA treatment response was lost. These results suggested that *MKK7* (−149/+172)-luc might be a bona fide reporter for monitoring MKK7 promoter activity and that the region of the *MKK7* promoter spanning −149 to −3 correlates with *MKK7* expression and the response to HDACIs. Taken together, these results indicated that HDACIs caused transcriptional suppression of MKK7 expression.

### Elevated MKK7-JNK activity antagonized HDACI-mediated suppression of c-Jun

**Figure 8:**
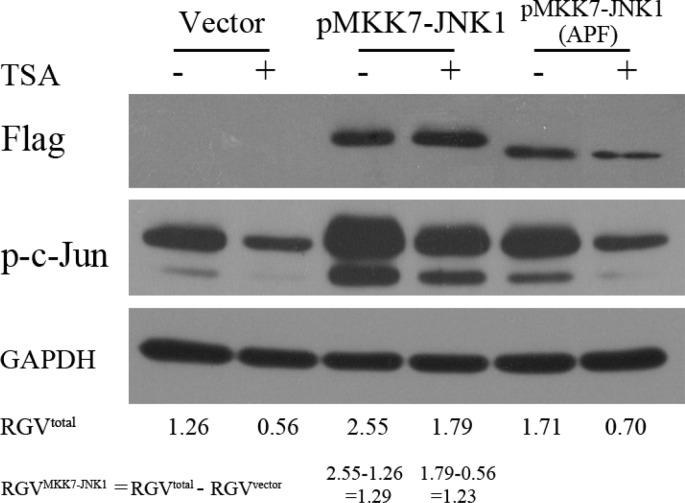
Elevated MKK7-JNK activity antagonized HDACI-mediated suppression of c-Jun SH-SY5Y cells transfected with vector and plasmids expressing MKK7-JNK1 or mutant MKK7-JNK1 (APF) for 24 hours were subjected to DMSO or TSA treatment for 12 hours, and then WB was performed with antibody against Flag and c-Jun. GAPDH was reprobed to verify equal loading. The raw gray value for p-c-Jun and GAPDH were acquired by using Image J software (National Institutes of Health) and the relative gray value (RGV) of p-c-Jun for each treatment group was calculated by the utility of the formula: Relative gray value of p-c-Jun = Raw gray value of p-c-Jun/Raw gray value of GAPDH.

To determine whether elevating MKK7-JNK activity was sufficient to antagonize HDACI-mediated suppression of c-Jun, we overexpressed a fusion protein, MKK7- JNK1, which was shown to exhibit markedly increased JNK activity compared with JNK1 or MKK7 alone. As shown in Figure 8, MKK7-JNK1 expression robustly increased c-Jun activation. Calculating the relative gray value for the phosphorylation of c-Jun exclusively evoked by ectopic MKK7-JNK1 in DMSO or TSA group, there was no obvious difference for the two values, suggesting that elevating MKK7-JNK activity efficiently rescued TSA-induced suppression on c-Jun activity. Expression of the MKK7-JNK1 (APF) fusion protein, a mutant of MKK7-JNK1 with a point mutation that catalytically inactivates JNK1, (tripeptide dual-phosphorylation motif Thr-Pro-Tyr replaced with Ala-Pro-Phe), largely lost the capacity in inducing c-Jun activity or rescuing TSA-mediated suppression.

Taken together, these results indicated that HDACIs suppressed c-Jun expression and activity through transcriptionally downregulating MKK7 and subsequently decreasing JNK activity.

### HDACI-induced MAPK phosphatase-1 (MKP-1) contributes to inactivation of JNK but not ERK

**Figure 9:**
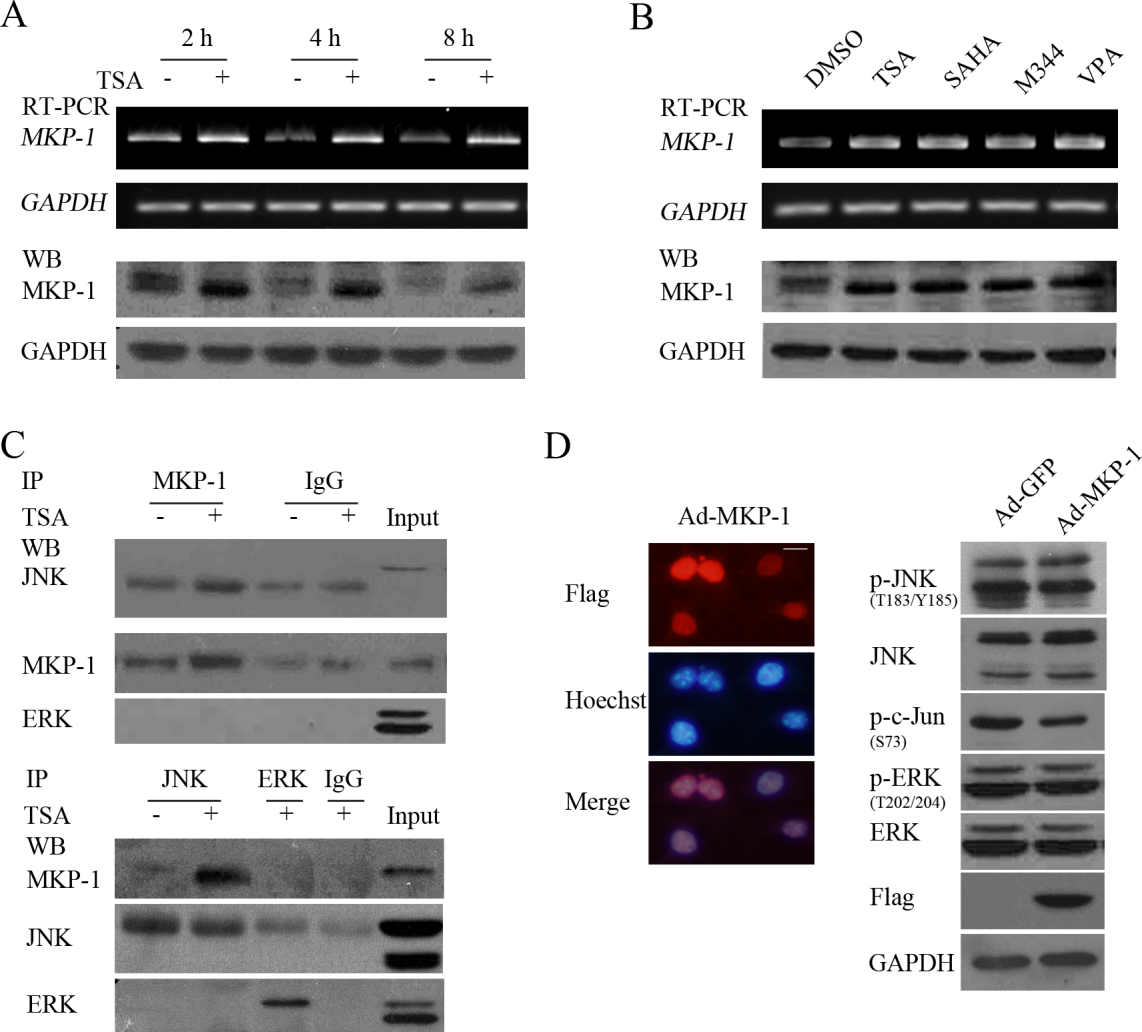
HDACIs-upregulated MKP-1 dephosphorylates JNK but not ERK (**A** and **B**) SH-SY5Y cells treated with 0.5 μM TSA for 2, 4 or 8 hours, or treated with HDACIs including TSA, SAHA, M344 and VPA for 2 hours were subjected to RT-PCR or WB for testing MKP-1 expression in both mRNA and protein levels. GAPDH was test to verify equal loading. (**C**) SH-SY5Y treated with 0.5 μM TSA for 4 hours were subjected to Co-Immunoprecipitation and following WB was performed with antibody against MKP-1, JNK and ERK. (**D**) SH-SY5Y cells infected with 100 MOI Ad-MKP-1 or Ad-GFP for 48 hours and following IF to determine their infectious efficiency (scale bar = 10 μm), WB was performed with antibody against phosphor-JNK, JNK, phosphor-ERK, ERK, phosphor-c-Jun and Flag (MKP-1 tag). GAPDH was reprobed to verify equal loading.

In mammalian cells, MKP-1 is the primary phosphatase responsible for dephosphorylation/deactivation of all MAPK members, including ERK, JNK and p38 [27]. We then determined whether HDACI-induced MKP-1 was involved in inactivating ERK and JNK in NB cells. MKP-1 was robustly induced by HDACI treatment at both the mRNA and protein levels in the early stage, earlier than 2 hours post-TSA treatment (Figure 9A and 9B). Co-IP assay indicated that MKP-1 preferentially interacts with JNK rather than ERK (Figure 9C). Moreover, adenovirus-mediated overexpression of MKP- 1 slightly decreased JNK/c-Jun phosphorylation but not ERK activities (Figure 9D). The results suggested that HDACI-induced MKP-1 contributes to inactivation of JNK instead of ERK, consistent with the previous reports in other cell types [28–30].

### Suppression of tumor growth and MKK7/c-Jun and Raf-1/Fra-1 activities in HDACI-treated SH-SY5Y xenografts

**Figure 10:**
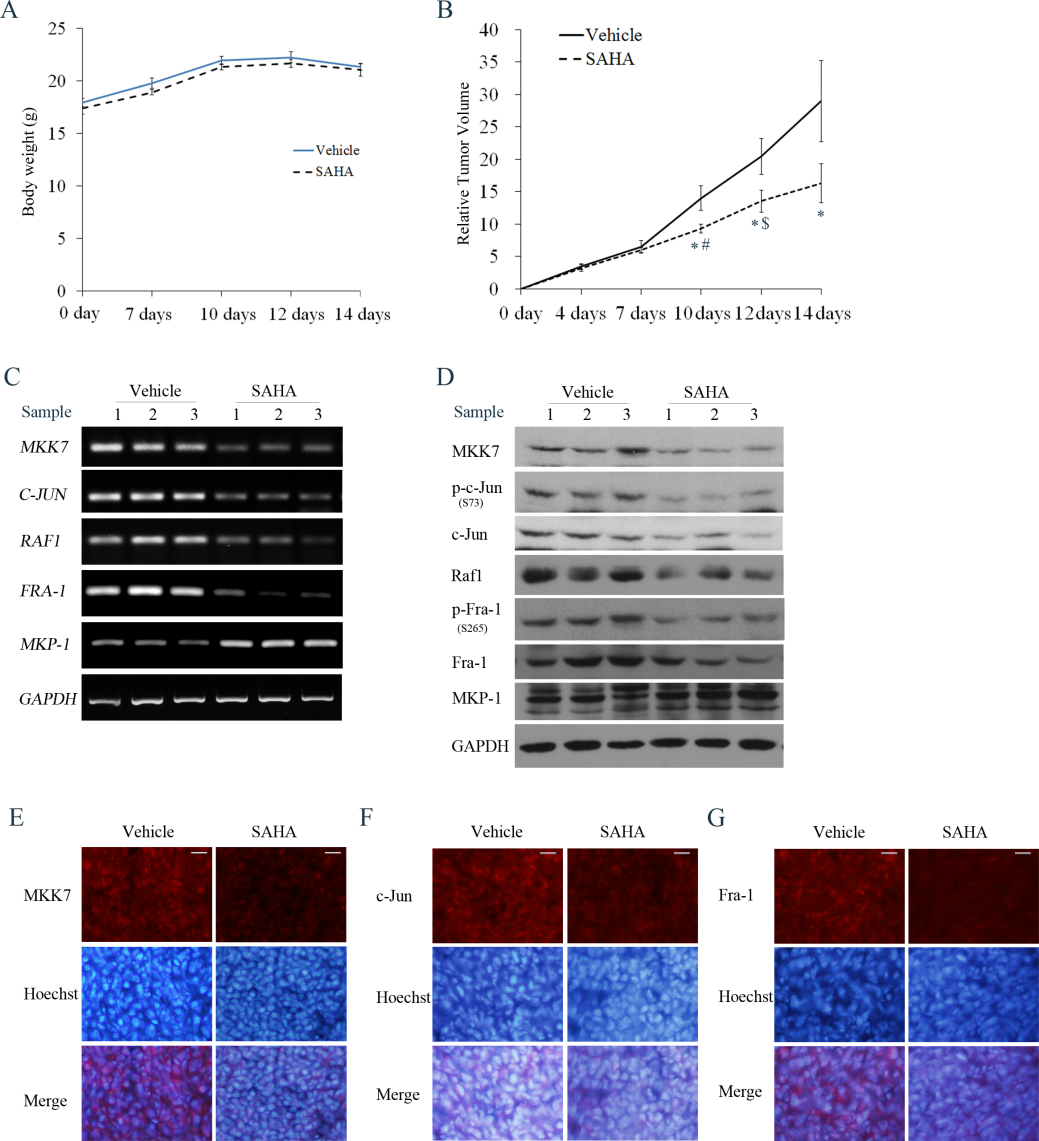
Effects of SAHA on tumor growth and MKK7/c-Jun and Raf1/Fra-1 expression in SH-SY5Y xenograft models Mice transplanted with SH-SY5Y xenografts were randomly divided into two groups (*n* = 6 per group) and treated every day for 14 days with vehicle or SAHA (50 mg/kg, i.p.). (**A**) The mean body weight of each group is expressed as the mean ± S.E. (*n* = 6 per group). (**B**) Tumor volume was measured at the indicated days after injection. Relative tumor volume (RTV) wascalculated as indicated in the Materials and methods, and the RTV is expressed as the mean ± S.E. (*n* = 6 per group); One-way ANOVA with selected pairs analysis, *P < 0.05 (Vehicle vs. SAHA), #*P* < 0.05 (SAHA at 7 days vs. SAHA at 10 days), $*P* < 0.05 (SAHA at 10 days vs. SAHA at 12 days). (**C**) Total RNA was extracted from the tumor tissues and RT-PCR was performed to test MKK7, Raf1, c-Jun, Fra-1 and MKP-1 mRNA levels. (**D**) Cell lysates extracted from tumor tissues from 3 mice in the vehicle or SAHA treatment groups were subjected to WB with antibody against MKK7, phosphor-c-Jun, c-Jun, Raf1, phosphor-Fra-1, Fra-1 and MKP-1. (**E**, **F** and **G**) IF was performed to analyze the expression of MKK7, c-Jun or Fra-1 in Vehicle and SAHA-treated samples (scale bar = 20 μm).

To further assess the anticancer effect of HDACIs, we investigated the *in vivo* efficacy of SAHA in an SH-SY5Y xenograft model. As shown in Figure 10A, no changes in animal body weight were observed between control and the SAHA group at any day analyzed (*P* > 0.05), suggesting that SAHA treatment did not cause obvious toxicity in nude mice. At 7 days post-treatment, SAHA treatment failed to significantly reduce the relative tumor volume (RTV) compared with the control (*P* > 0.05). However, at 10 days post-treatment, SAHA exerted a significant inhibitory effect on tumor growth (*P* < 0.05) and caused greater inhibition at 12 or 14 days post-treatment (*P* < 0.05, (Figure 10B and Table 1). These results demonstrated that SAHA administration led to a remarkable inhibition of SH- SY5Y tumor xenograft growth.

**Table 1:** *In vivo* efficacy of SAHA against SH-SY5Y xenografts (mean ± S.D.)

	Animals		Body weight (g)		Tumor volume (mm^3^)		Mean RTV	TGI (1-T/C)%	IR (%)
Group	Start	End	Start	End	Start	End			
Vehicle	6	6	19.77 ± 0.48	21.27 ± 0.33	82.34 ± 2.04	2369.13 ± 428.66	29.00		
SAHA	6	6	19.23 ± 0.51	20.93 ± 0.55	80.47 ± 1.68	1322.88 ± 110.51	16.31	43.76	41.68

RTV: relative tumor volume

TGI: tumor growth inhibition

T/C: mean RTV of treatment/mean RTV of Vehicle

IR: inhibition rate

We next clarified whether both MKK7/c-Jun and Raf1/Fra-1 inactivation, and MKP-1 induction occurred in the SAHA-treated xenograft model. RT-PCR results showed exposure to SAHA induced an obvious decrease in MKK7, Raf-1, c-Jun and Fra-1 mRNA levels, and an increase in MKP-1 mRNA levels (Figure 10C). Consistently, SAHA administration caused a decrease in MKK7 and Raf-1 protein expression, c-Jun and Fra- 1 phosphorylation levels, and an increase in MKP- 1 protein expression (Figure 10D). IF demonstrated that the immunostaining signals of MKK7, c-Jun and Fra- 1 in SAHA-treated slices were lower than those in control slices (Figure 10E–10G). Data for Raf-1 and MKP-1 immunostaining could not be obtained due to strong background signals. Taken together, these results highlighted that suppression of both MKK-7/c-Jun and Raf-1/Fra-1 activities was involved in the tumor-growth inhibitory effects exerted by SAHA *in vivo*, in agreement with the *in vitro* data.

## DISCUSSION

In this study, we identified that c-Jun primarily dimerization with Fra-1 promotes the proliferation of both MYCN single copy and MYCN amplified NB cells and that HDACIs can transcriptionally suppress c-Jun and Fra- 1 expression. Mechanistically, HDACIs suppress c-Jun and Fra-1 expression through transcriptionally downregulating MKK-7 and Raf-1 and subsequently decreasing JNK and ERK activities. Our *in vitro* and *in vivo* findings highlighted a new mechanism of HDACI action, in that HDACIs can suppress AP-1 oncogenes by selectively inactivating their upstream cascades in tumor cells.

However, c-Jun is a well-known transcriptional factor with Janus roles in regulating cell fate, and previous studies have demonstrated that c-Jun exhibits a proapoptotic function in NB cells [31, 32]. Notably, Waetzig and colleagues reported that basal endogenous c-Jun, which is essential for cellular viability, could transfer to induce apoptosis under stressful conditions in SH-SY5Y cells but that the c-Jun upstream kinase remains JNK2 [33]. These interesting observations suggested that c-Jun, not JNK, plays a decisive role in enabling NB cells to survive or die, differing from a previous observation in neurons that JNK signals from different pools contribute to distinct c-Jun roles [34]. The hypothesis that c-Jun in association with different partners regulates a diverse set of target genes, thus exhibiting distinct roles, might be a reasonable explanation for this action. Indeed, alterations in AP-1 composition have been associated with cell fate or phenotype changes in cerebellar granule neurons [35], glioma cells [36] and human melanoma [37] following stress treatment. Therefore, specific partners play a decisive role in c-Jun-controlled cell fate.

The Fra-1/Jun dimer exhibits versatile roles in cancer formation and progression [38–40] and is considered determinants of tumor heterogeneity by retaining tumor cell plasticity and clonal selection within a tumor [41, 42]. Based on our results, HDACIs are effective tools in targeting the oncogenic roles of c-Jun/Fra-1 via suppressing their expression in both MYCN single and amplified NB cells. However, in several tumor cell lines such as human leukemia cells [43] and colon cancer cells [44] JNK/c-Jun is activated by HDACIs to promote apoptosis, and HDAC inhibition-induced ERK activation contributes to early M-phase (prometaphase) arrest and subsequent apoptosis in prostate cancer cell LNCaP [45]. By increasing the concentration of TSA or the exposure duration of SH-SY5Y and SK-N-SH cells to TSA, we did not detect JNK/c-Jun or ERK/Fra-1 activation, although typical caspase-dependent apoptosis occurred. Moreover, all HDACIs displayed similar effects in inhibiting JNK/c-Jun and ERK/Fra-1 activities. Our results indicated that the selective suppression of oncogenic AP-1 proteins and upstream kinases is involved in HDACI-mediated anticancer effects.

HDACI-mediated suppression on c-Jun/Fra-1 is derived from their inhibition on MKK7 and Raf-1 and consequent inactivation of JNK and ERK. To the best of our knowledge, this study is first to demonstrate that *MKK7* is an inducible gene and that its expression is tightly dependent on HDAC activity, although the exact mechanism requires further exploration. MKK7 and Raf-1 are two critical kinases in the transfer of signals from upstream kinases such as RhoA/cdc42 [44] and Ras [46], which are commonly activated by extrinsic stimuli, to intrinsic JNK/Jun and ERK/Fos cascades, respectively. Aberrant Ras/Raf/MEK/ERK or RhoA/cdc42/MLKs/MKK/JNK cascades have been implicated in many types of diseases such as tumor formation [47] and neurodegenerative diseases [48]. Thus, HDACI-mediated suppression of the expression of these two kinases might be a promising strategy for treating or preventing these diseases.

Several recent reports have stated that induced MAPK phosphatase-1 (MKP-1) contributes to HDACI-mediated inactivation of JNK [28–30]. However, adenovirus-mediated overexpression of MKP-1 only slightly decreased JNK/c-Jun phosphorylation compared with the severe inactivation of JNK activities induced by MKK7 knockdown (Figure 9). Furthermore, MKP-1 was robustly induced earlier than 2 hours post-TSA treatment, whereas remarkable JNK dephosphorylation occurred at 8 hour post-HDACI treatment, later than the downregulation of MKK7 at 4 hours post-TSA treatment (Figure 7). These results suggested that MKK7 downregulation might be a precondition of MKP-1-mediated inactivation of JNK activity upon HDACI treatment. This hypothesis was supported by recent studies demonstrating that the conserved KIM-motif (kinase interaction motif) of MAPK kinases essential for MAPK regulator binding to the docking site in the C-terminal lobe of MAPKs differs from that of MKPs. MKKs, including MEK1/2, MKK3/6, MKK4/7, and STE7, possess a flexible N-terminal extension containing a linear 15 amino acid KIM. In contrast, in MKPs, the KIM is not a linear peptide but part of the structured MAPK binding domain (MKBD), which results in more constraints in the selectivity and capacity of MKP binding to MAPKs than MKKs [49]. Moreover, MKP binding to MAPK leads to a conformational change of the C-terminal catalytic domain, which is crucial for enzymatic activation of MKP [50]. Taken together, HDACI-mediated downregulation of MKK7 not only decreases the kinase signal in phosphorylating JNK but also enable MKP-1 to bind enzymatic activation, inactivating JNK/c-Jun cascades.

**Figure 11:**
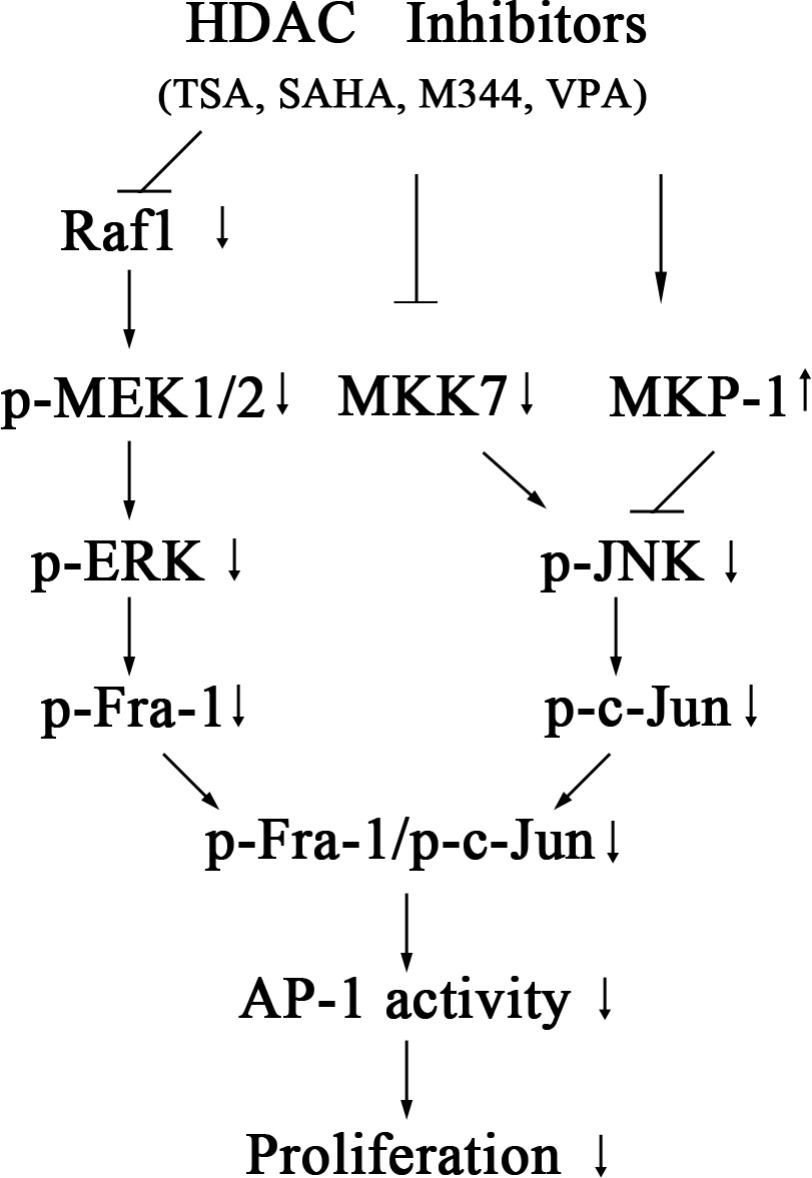
Model for the signaling pathways involved in HDACI-induced suppression of c-Jun/Fra-1-mediated AP-1 activity and proliferation in NB cells Exposure to HDACIs (TSA, SAHA, M344 andVPA) causes transcriptional upregulation of MKP-1 and downregulation of both MKK7 and Raf-1. The HDACI-induced decrease in Raf-1 leads to the inactivation of MEK/ERK/Fra-1 cascades, whereas the HDACI-mediated decrease in MKK7 and increase in MKP-1 synergistically inactivate JNK/c-Jun activities. c-Jun/Fra-1-mediated AP-1 activity promotes proliferation of NB cells.

In conclusion, the c-Jun/Fra-1 dimer promotes the proliferation of NB cells, and exposure to HDACIs (TSA, VPA, M344 and SAHA) can effectively suppress c-Jun/Fra-1 expression through the transcriptional upregulation of MKP-1 and downregulation of both MKK7 and Raf-1. HDACI-induced decreased Raf-1 expression leads to the inactivation of MEK/ERK/Fra-1 cascades, whereas the HDACI-mediated decrease in MKK7 and increase in MKP-1 synergistically inactivate JNK/c-Jun activities (Figure 11). The findings suggested that administration of HDACI might be a promising trial in treating NB patients and two FDA-proved HDACI drugs VPA and SAHA are in hands. Furthermore, this study provides new insight into the molecular mechanism of the anti-tumor selectivity of HDACIs and suggests that HDACIs might be a more effective clinical target in tumors with high c-Jun/Fra-1 activity mediated by MKK7 and Raf1.

## MATERIALS AND METHODS

### Cell culture and drug treatments

NB cells with MYCN single copy SH-SY5Y, SK-N-SH and NB cells with MYCN amplified SK-N-BE(2), KP-N-NS were obtained from the Type Culture Collection of the Chinese Academy of Sciences (Shanghai, China) and cultured in whole media containing DMEM with 2 mM L-glutamine, 4.5 g/l glucose, 10% fetal bovine serum (Hyclone) and 1% penicillin-streptomycin (10,000 U/ml, Invitrogen, USA). TSA, VPA sodium (VPA Na), M344 and GW5074 were purchased from Sigma-Aldrich. SAHA was purchased from Selleck Chemicals. SP600125 and U0126 were purchase from Calbiochem. Each of these inhibitors was added to media when cells grew to 60–70% confluence.

### Cell viability and proliferation assay (MTT assay)

Cells were seeded in 96-well plates at 1 × 10^3^ per well in 150 μl media. After the cells were treated, MTT (Sigma-Aldrich) was added to each well at a final concentration of 0.5 mg/ml and incubated for 4 h at 37°C. Then, the medium was removed, and formazan crystals were dissolved in 150 μl DMSO. Absorbance was measured at 570 nm using a spectrophotometer (Tecan Infinite, Swiss). Cell numbers were calculated based on a standard curve derived from serial cell dilution.

### Colony formation assay

A colony formation assay was used to detect the ability of tumor cells to grow in soft agar independent of anchorage, which considered the most stringent assay for detecting malignant transformation of cells. Briefly, to detect the ability of cells to grow in soft agar independent of anchorage, 0.65% soft agar (w/v) melt in whole media was prepared and added to 6-well plates (1.5 ml/well) as the bottom layer. During solidification of the bottom laser at 37°C, the pretreated cells were trypsinized, collected in whole media and then mixed with the melt 0.65% agar media at a ratio of 1:1 as a cell layer containing 1 × 10^3^ cells/ml. Then, 1 ml cell layer media was pipetted on the solidified bottom layer, and a top layer (1 ml, 0.65% agar media) was added until the cell layer became solidified. To observe the effects of inhibitors on colony formation, the top layer was replaced with whole medium containing either the indicated inhibitor or 0.1% solvent control and changed twice per week. To observe the effect of dominant negative c-Jun (c-JunDN) on colony formation, SH-SY5Y cells were infected with Ad-c-JunDN or Ad-GFP (MOI 100). Twenty hours later, the infected cells were split and seeded at 1 × 10^3^ cells/ml. Cells were incubated in 5% CO_2_ at 37°C for 14 days, and colonies were fixed and stained with 0.005% crystal violet in methanol. Triplicate wells were used for each treatment, and three independent experiments were performed. The number of colonies with a diameter > 50 μm was counted under a microscope at 40 × magnification, and colony forming efficiency was determined as the percentage of plated cells that formed colonies.

### Reverse transcription-PCR (RT-PCR)

Total RNA was extracted and isolated from cells using TRIzol reagent (Invitrogen), and reverse transcription was performed to obtain cDNA as described previously [35]. Gene-specific primers to detect the expression levels of c-Jun, Fra-1, Raf1, MKK7 and MKP-1 were designed using Primer Premier 5.0 software and are listed in Supplementary Data S1. The housekeeping gene GAPDH was amplified as a normalization control.

### Western blot (WB) analysis

WB analyses were performed as described previously [35]. Primary antibodies against p-Fra-1 (Ser256), Fra-1, p-c-Jun (Ser73), c-Jun, MKP-1, p-JNK (Thr183/185), JNK, p-ERK (Thr202/204), ERK, Raf1, MKK7, p-MKK4 (Ser257/Thr261), MKK4, MEK1/2, p-MEK1/2 (Ser217/221), Flag and GAPDH are listed in Supplementary Data S1.

### Immunofluorescence (IF) and BrdU incorporation

IF was performed as described previously [35]. The antibodies used in these studies included mouse anti-c-Jun (BD Technology), rabbit anti-Fra-1 (Cell Signaling Technology) and mouse anti-Flag (Sigma-Aldrich) antibodies. CY3- and FITC-conjugated secondary antibodies (Jackson ImmunoRes) were used, and confocal imaging were performed as described previously [35].

For the BrdU incorporation assay, cells were seeded in 24-well plates, and BrdU (20 μM final concentration, Sigma-Aldrich) was added to culture media 6 hours before the cells were fixed in 4% paraformaldehyde (PFA) in phosphate-buffered saline (PBS). IF labeling was then performed using mouse anti-BrdU (1:400) (Sigma-Aldrich) according to standard procedures. Hoechst 33258 (Sigma-Aldrich) was used to stain nuclei. Images of at least six random fields were acquired, and the incorporation ratio of BrdU was determined as the percentage of nuclei that co-stained for BrdU.

For tumor samples, tumors were immersed in OCT compound (Sakura, USA) and frozen at −80°C. The sections (4 μm) were permeabilized with 0.25% Triton X-100 in PBS and blocked with 10% donkey antiserum dissolved in 0.25% Triton X-100 and PBS to avoid nonspecific binding for 60 minutes and subsequently incubated with rabbit anti-MKK7 (1:400), rabbit anti-Raf1 (1:400), rabbit anti-phospho-c-Jun(Ser73)(1:400) or rabbit anti-phospho-Fra-1(Ser265)(1:400) antibodies. The sections were washed and further incubated with the corresponding CY3-conjugated secondary antibodies, and nuclei were stained with Hoechst 33258. Images were acquired using an Olympus inverted microscope (IX-71).

### Immunoprecipitation (IP) assays

IP assays were performed as described previously [35]. For each trial, cell extracts were immunoprecipitated with 2 μg each antibody and incubated with 30 μl agarose hydrazide beads with protein A plus G (Calbiochem), and the immunocomplexes were subjected to WB analysis. The antibodies used in the experiments against c-Jun, Fra-1, ATF2, Fra2, c-Fos, FosB, JunB, JunD are listed in Supplementary Data S1.

### Histone extraction

Briefly, cells were washed twice with ice-cold PBS supplemented with 5 mM sodium butyrate and then suspended in Triton extraction buffer (TEB: PBS containing 0.5% Triton X-100 (v/v), 2 mM phenylmethylsulfonylfluoride (PMSF), and 0.02% (w/v) NaN_3_). The cell lysate was centrifuged at 6,500 × g for 10 minutes at 4°C to spin down the nuclei, and the pellets were re-suspended in 0.2 N HCl overnight at 4°C for acid extraction of the histones. After the samples were centrifuged, the supernatant that contained histone protein was saved for further analysis.

### Gel mobility shift assay

Nuclear extracts were prepared as described in detail previously [35]. AP-1 probes containing the conserved TRE sequence (TGACTCA, AP-1 site) as underlined (forward: 5′-CGCTTG ATGACTCAGCCGGAA-3′ and reverse: 3′-GCGAACTACTGAGTCG GCCTT-5′) and mutated AP-1 probes in which the conserved TRE sequence TGACTCAwasmutated to TGACTTG were annealed and labeled with γ^32^P (Perkin Elmer Life and Analytical Sciences) using T4 polynucleotide kinase. ^32^P-labelled probes were incubated with 5 mg nuclear protein in 20 μl DNA binding reaction buffer. For supershift, 1 μg c-Jun (sc-1694×, Santa Cruz) or Fra-1 antibody (sc-183, Santa Cruz) was preincubated with nuclear extracts at 4°C for one hour. DNA-protein complexes were resolved by 4% polyacrylamide gel and imaged.

### Plasmids, reporters, and adenovirus vectors

Plasmids containing Raf1 and its mutants Raf1 (50–151) and Raf1 (50–220) were a kind gift from Dr. Tomas Balla (Eunice Shriver Kennedy National Institute for Child Health and Human Development, National Institutes of Health, USA) [51]. The reporter plasmids MMP-1-luciferase and MMP-1 (AP-1 mutant)-luciferase were a kind gift from Dr. Ralf Janknecht (Health Sciences Center, University of Oklahoma, USA) [52]. The plasmids c-Jun, c-JunDN, and 7 ×AP-1-luc were described previously [35]. The MKP1- or Fra-1-expressing plasmids were constructed by cloning the coding sequence into the pShuttle vector (Stratagene), and adenoviruses expressing c-JunDN, the JNK binding domain (JBD), c-Jun, Fra-1 or MKP1 were generated as described previously [19]. To construct an original MKK7 reporter, a fragment spanning from −857 to + 172 relative to the transcription start site of the human *MKK7* genomic sequence was produced by PCR with the forward primer 5′-GCACCCGTCGGACCACTTAAAGGAG-3′ and the common reverse primer 5′-GGGCTGATA TCCAGGTTGAGGTCGA-3′. This fragment was fused to the promoter-less-firefly luciferase gene of the pGL3-Basic vector (Promega) to generate an *MKK7* (−857/+ 172)-luc reporter. A series of reporter constructs with the same 3′-end but with different 5′-ends was also constructed by PCR using *MKK7* (−857/+ 172)-luc as the template and the common above-mentioned reverse primer. The forward primers used were as follows: 5′-GCTGGCAAGAAGGGAAAGGGCTCTC-3′ for *MKK7* (−575/+ 172)-luc, 5′-ACCGTGAAAAGCGAGGAGGC TGAGG-3′ for *MKK7* (−420/+ 172)-luc, 5′-CAGAAGA ATGGTGTTTCCTCGCAGC-3′ for *MKK7* (−258/+ 172)-luc, 5′-TCGAGCTCTAGG TGGCGTCATCCTT-3′ for *MKK7* (−149/+ 172)-luc, 5′-AGTGCGGTGTTTGTCTG CCGGACTG-3′ for *MKK7* (−3/+ 172)-luc, and 5′-GTCC TCCCTGGAACAGAAGC-3′ for *MKK7* (+ 74/+ 172)-luc.

### Transient transfection, small RNA interference and luciferase assays

For the transfection of siRNAs or expression plasmids into SH-SY5Y cells, electrotransfection was performed using an Amaxa machine (Lonza) according to the manufacturer's protocol (program T-024). Small interfering RNAs (siRNAs) targeting c-Jun, Fra-1, MKK7, and MKK4 are listed in Supplementary Data S1. For transfection of siRNAs or expressing plasmids into SK-N-SH, SK-N-BE (2) and KP-N-NS cells, jetPRIME transfection reagent (Polyplus-transfection, France) was used according to the manufacturer's instructions. For luciferase assays, cells plated in 24-well clusters were transfected with 550 ng plasmid/well, including 400 ng reporter plasmid, 50 ng *Renilla* luciferase (RL) reporter (pCMV-RL) and 100 ng expression plasmids or mock DNA vectors using jetPRIME transfection reagent. Dual reporter assays were performed as described previously [35].

### Tumor xenografts and anti-tumor effect evaluation *in vivo*

All experimental protocols for tumor xenografts were approved by the Animal Care and Use Committee of Guangzhou Medical University. Human SH-SY5Y xenografts were established by subcutaneously inoculating 5 × 10^6^ cells into BALB/c nude mice (4–5 weeks old). When the tumors reached a size of 1500–2000 mm^3^, the tumors were removed from anesthetized mice and cut into 4 × 4 × 4 mm blocks. The small blocks were implanted into new nude mice, and the mice were randomized into control and treatment groups when the tumors reached a mean group size of 80–90 mm^3^. The following treatments were administered every day for 14 days: control group: vehicle (1% dimethyl sulfoxide (DMSO), 7% cremophor/ethanol (3:1) and 92% PBS, intraperitoneally (i.p.)); treatment group:SAHA (50 mg/kg, i.p.). Tumor volume (V) was calculated as V = (length × width^2^)/2. The individual relative tumor volume (RTV) was calculated according to the following formula: RTV = Vn/V0, where Vn is the tumor volume on day n and V0 is the tumor volume on the first day of treatment. Drug efficacy was expressed as the percentage of tumor growth inhibition (TGI), which was calculated using the equation (1 – T/C) × 100%, where T is the mean RTV of the treated tumor and C is the mean RTV in the control group. The tumor inhibition rate was calculated using the equation (1 – Wt/Wc) × 100%, where Wc is the mean weight of control tumor and Wt is the mean weight of treatment group.

### Statistical analysis

All immunoblots illustrate representative results from at least three experiments, and all other experiments were repeated at least three times. The results are expressed as the mean ± SE. Comparisons were analyzed with two-way ANOVA or one-way ANOVA with selected pairs analysis. *P* < 0.05 was considered significant.

## SUPPLEMENTARY MATERIALS FIGURES


