# Supplementary material for: HDAC inhibitors suppress c-Jun/Fra-1-mediated proliferation through transcriptionally downregulating MKK7 and Raf1 in neuroblastoma cells
Source: Oncotarget. 2015 Dec 30;7(6):6727–47. doi: 10.18632/oncotarget.6797 (PMC4872745; doi:10.18632/oncotarget.6797)
Supplement: Supplementary file 1 [file oncotarget-07-6727-s001.pdf]

HDAC inhibitors suppress c-Jun/Fra-1-mediated proliferation through transcriptionally downregulating MKK7 and Raf1 in neuroblastoma cells

Supplementary Materials

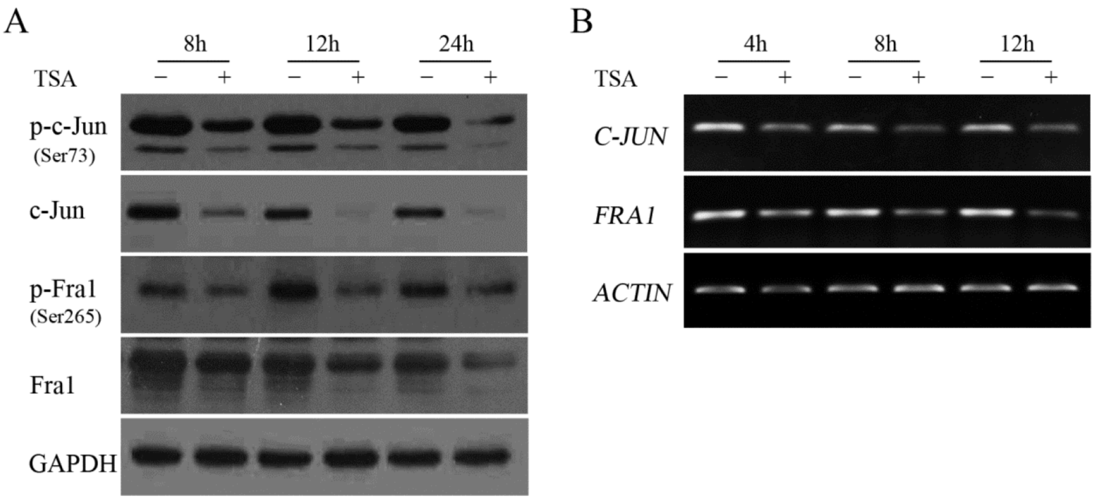

Supplementary Figure S1: HDACI caused a transcriptional suppression on c-Jun and Fra1 in SK-N-SH. (**A**) SK-N-SH cells were treated 0.5 µM TSA for 8 h, 12 h and 24 h, following WB was performed to detect expression and phosphorylation of c-Jun, Fra1. GAPDH was reprobed to verify equal loading. (**B**) SK-N-SH cells were treated 0.5 µM TSA for 4 h, 8 h and 12 h and total mRNA was extracted and subjected to RT-PCR with specific primers against c-Jun and Fra1. GAPDH was amplified to verify equal input.

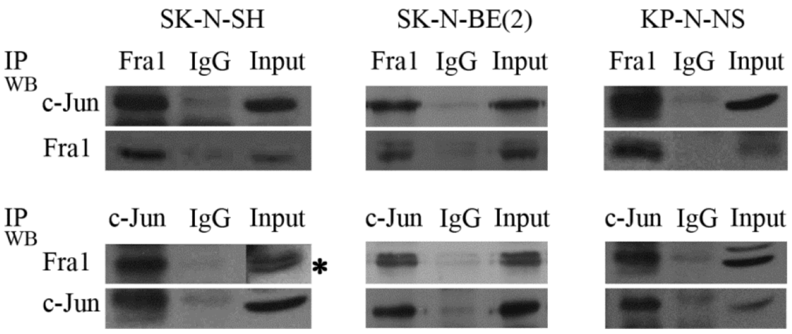

Supplementary Figure S2: c-Jun interacts with Fra1 as a dimer in both MYCN single copy and MYCN amplified NB cells. SK-N-SH, SK-N-BE(2) and KP-N-NS cells were lysed for Immunoprecipitation (IP) assays and c-Jun or Fra1 was detected by WB in the precipitates pulled down by antibody against c-Jun, Fra1 or normal IgG. Input means the cellular lysis before adding antibody. * The signal of Fra1 appeared in the "Input lane" was resulted from a long time of exposure.

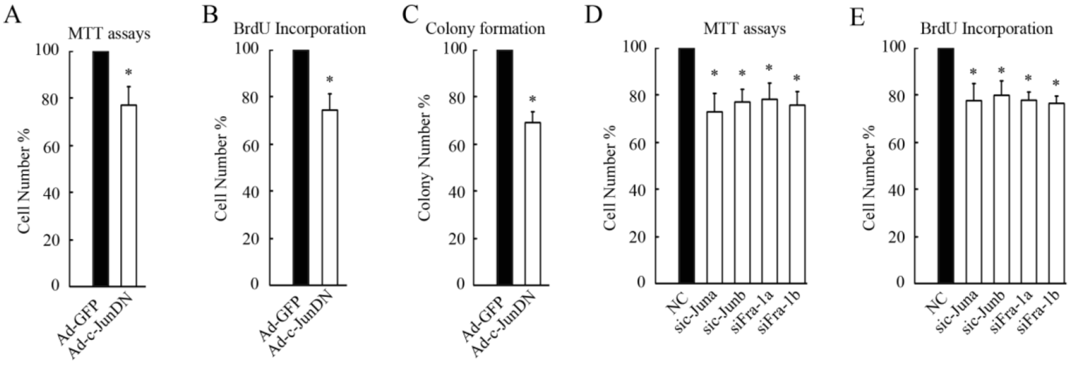

Supplementary Figure S3: c-Jun/Fra-1 dimer-mediated TRE activity is essential for SK-N-SH cell proliferation. (**A** and **B**) SK-N-SH cells were infected with 100 MOI Ad-c-JunDN or control Ad-GFP for 48 hours, and MTT assays and BrdU incorporation were performed to determine proliferation rates. The data are presented as the mean ± S.E. $n = 3$; One-way ANOVA analysis, *$P < 0.05$ (Ad-GFP vs. Ad-c-JunDN). (**C**) SK-N-SH cells infected with Ad-c-JunDN or Ad-GFP (MOI 100) were seeded onto plates at 11000 cells/ml for colony formation assays. The number of colonies with a diameter > 50 µm was counted under a microscope, and images were acquired at 40× magnification. The data are presented as the mean ± S.E. $n = 3$; One-way ANOVA analysis, *$P < 0.05$ (Ad-GFP vs. Ad-c-JunDN). (**D** and **E**) SK-N-SH cells were transfected with non-specific siRNA control (NC), sic-Juna, sic-Junb, siFra-1a or siFra-1b for 48 hours, and following MTT assays and BrdU incorporation were performed to determine proliferation rates. The data are presented as the mean ± S.E. $n = 3$; One-way ANOVA analysis with selected pairs, *$P < 0.05$ (NC vs. siRNA).

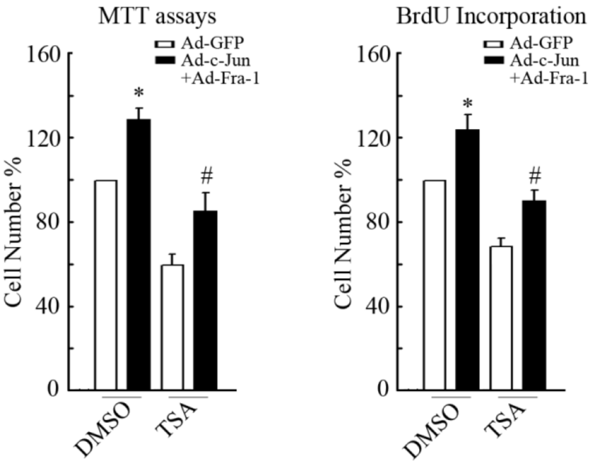

Supplementary Figure S4: Co-overexpression of c-Jun and Fra-1 promoted proliferation and antagonized HDACI-mediated inhibitory effects in SK-N-SH cells. SK-N-SH cells were infected with Ad-c-Jun + Ad-Fra-1, and Ad-GFP was included as control. Forty-eight hours later, infected cells were treated with 0.5 µM TSA for 24 hours, MTT assays and BrdU incorporation was performed to determine proliferation rates. The data are presented as the mean ± S.E. $n = 3$; One-way ANOVA analysis, *$P < 0.05$ (DMSO: Ad-GFP vs. Ad-c-Jun + Ad-Fra-1); #$P < 0.05$ (TSA: Ad-GFP vs. Ad-c-Jun + Ad-Fra-1).

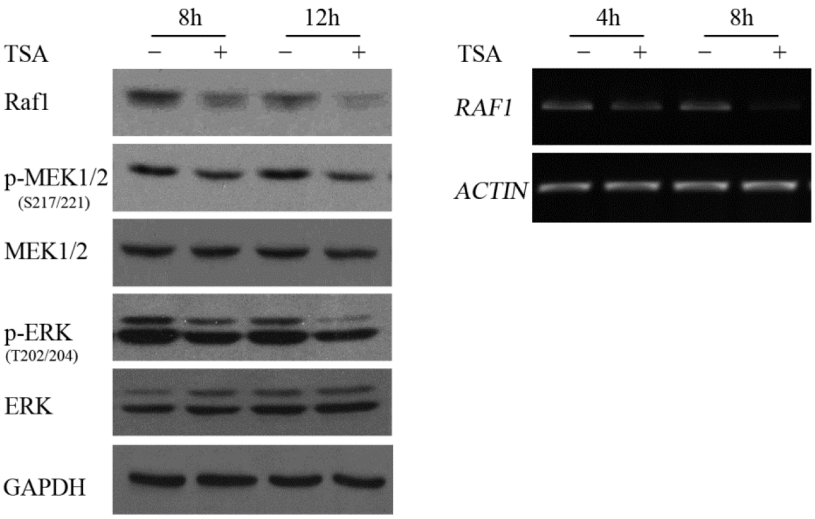

Supplementary Figure S5: HDACI transcriptionally suppressed Raf1 expression and subsequently decreased the activity of MEK1/2/ERK in SK-N-SH cells. (**A**) SK-N-SH cells were treated 0.5 µM TSA for 8 h, 12 h and 24 h, following WB was performed with antibody against Raf1, phosphor-MEK1/2, MEK1/2, phosphor-ERK or ERK. GAPDH was reprobed to verify equal loading. (**B**) SK-N-SH cells were treated 0.5 µM TSA for 4 h, 8 h and RT-PCR was performed with specific primers against *RAF1*. GAPDH was amplified to verify equal input.

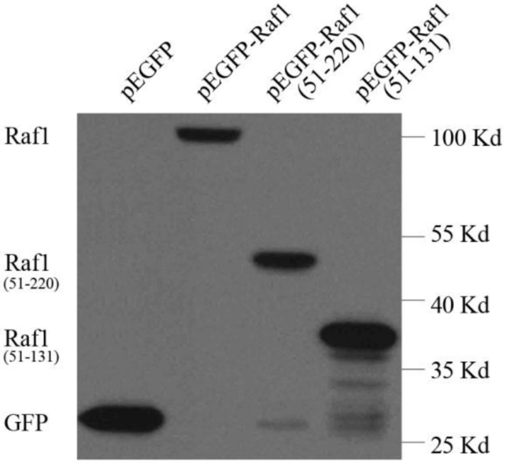

Supplementary Figure S6: Expression of plasmids Raf1 and mutants. SH-SY5Y cells were transfected vector or the plasmids expressing Raf1, Raf1 (51–131) and Raf1 (51–220) and following WB was performed with antibody GFP.

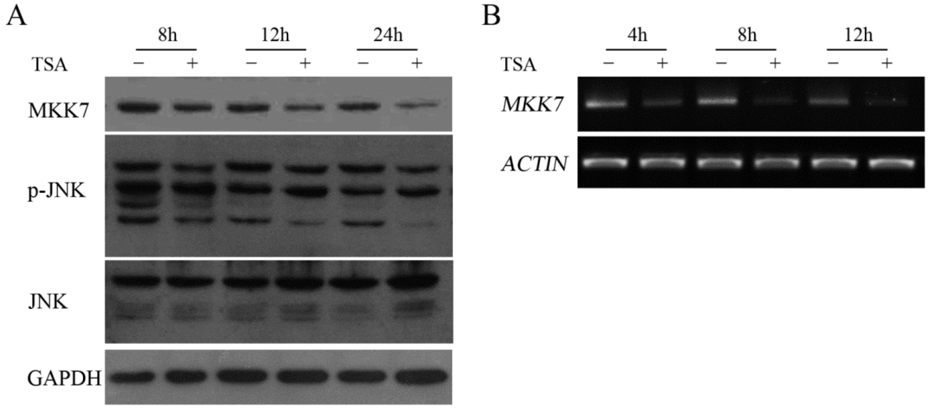

Supplementary Figure S7: HDACI transcriptionally suppressed MKK7 expression and subsequently decreased the activity of JNK in SK-N-SH cells. (**A**) SK-N-SH cells were treated 0.5 µM TSA for 8 h, 12 h and 24 h, following WB was performed with antibody against MKK7, phosphor-JNK or JNK. GAPDH was reprobed to verify equal loading. (**B**) SK-N-SH cells were treated 0.5 µM TSA for 4 h, 8 h and RT-PCR was performed with specific primers against *MKK7*. GAPDH was amplified to verify equal input.

MATERIAL AND METHODS

Antibodies

1. c-Jun (H79) Cat: sc-1694 Santa Cruz Biosciences (SCB)
2. c-Jun Cat: 610327 BD Biosciences (BD)
3. p-c-Jun(s73) Cat: 9164s Cell Signaling Technologies (CST)
4. p-FRA1(S265) Cat: 3880s CST
5. Fra-1(N-17) Cat: sc-183 SCB
6. MKK7, Cat: 1949-1 EPITMICS
7. p-MEK1/2/217/221) (41G9) Cat: 9154s CST
8. MEK1/2(1215) Cat: BS3600 Bioworld
9. Phospho-p44/42 MAPK(Erk1/2)(Thr202/Tyr204) (DB.14.4E) Cat: 4370 CST
10. p42/44 MAPK Cat: 9102 CST
11. Raf-1(E-10) Cat: sc-7267 SCB
12. P-SAPK/JNK(T183/Y185) Cat: 9251s CST
13. JNK(D2) Cat: sc-7345 SCB
14. SEK1/MKK4 Cat: 9152s CST
15. Phosphor-SEK1/MKK4 Cat: 4514s CST
16. Flag Cat: F3165 Sigma
17. GAPDH Cat:AG019 Beyotime

siRNAs

Fra1a: 5′-GAGUAAGGCGCGAGCGGAATT-3′
5′-UUCCGCUCGCGCCUUACUCTT-3′
Fra1b: 5′-GACUGACAAACUGGAAGAUTT-3′
5′-AUCUUCCAGUUUGUCAGUCTT-3′
c-juna: 5′-AGUCAUGAACCACGUUAACTT-3′
5′-GUUAACGUGGUUCAUGACUTT-3′
c-junb: 5′-GGCACAGCUUAAACAGAAATT-3′
5′-UUUCUGUUUAAGCUGUGCCTT-3′
mkk7a: 5′-CCAACACGGACGUCUUCAUTT-3′
5′-AUGAAGACGUCCGUGUUGGTT-3′
mkk7b: 5′-GCUGGCAACAGGACAGUUUTT-3′
5′-AAACUGUCCUGUUGCCAGCTT-3′
mkk4: 5′-GCCUUACGAAGGAUGAAUCCATT-3′
5′-UGGAUUCAUCCUUCGUAAGGCTT-3′

Primers for RT-PCR

c-Jun (118 bp):
Forward: 5′-CTCCAAGTGCCGAAAAAGGAAG-3′
Reverse: 5′-CACCTGTTCCCTGAGCATGTTG-3′

Fra1 (139 bp):
Forward:5′-GCCTGTGCTTGAACCTGA-3′
Reverse:5′-GCTGCTACTCTTGCGATGA-3′

Raf-1 (390 bp):
Forward: 5′-CGCTTAGATTGGAATACTGA-3′
Reverse: 5′-AAAGGTGAAGGCGTGAG-3′

MKK7 (201 bp)
Forward: 5′-AACAAGCGCATCCTCATGGAC-3′
Reverse: 5′-AATCGCCACTGTCATCTTGCC-3′

MKP1 (497 bp)
Forward: 5′-CTGCCGCTCCTTCTTCGCTTTC-3′
Reverse: 5′-TGTCCTTGCGGGAAGCGTGATA-3′

GAPDH
Forward: 5′-GCCACATCGCTCAGACAC-3′
Reverse: 5′-CATCACGCCACAGTTTCC-3′
